# Supplementary material for: Genome-wide identification, characterization and gene expression of BES1 transcription factor family in grapevine (Vitis vinifera L.)
Source: Sci Rep. 2023 Jan 5;13:240. doi: 10.1038/s41598-022-24407-y (PMC9816167; doi:10.1038/s41598-022-24407-y)
Supplement: Supplementary file 3 — Supplementary Information. [file 41598_2022_24407_MOESM3_ESM.zip › Vvi_Atr/Vitis_vinifera.PN40024.v4.dna_sm.toplevel.fa.vs.Amborella_trichopoda.AMTR1.0.dna_sm.toplevel.fa.html/Vvi-16.html]

|  |  |  |  |  |  |  |  |  |  |  |  |  |  |
| --- | --- | --- | --- | --- | --- | --- | --- | --- | --- | --- | --- | --- | --- |
| Duplication depth | Reference chromosome | Collinear blocks | | | | | | | | | | | |
| 1 | Vvi-Vitvi16g00002\_t001 |  | Atr-ERN10200 |  |  |  |  |  |  |  |  |  |  |
| 1 | Vvi-Vitvi16g00003\_t001 |  | Atr-ERN10208 |  |  |  |  |  |  |  |  |  |  |
| 1 | Vvi-Vitvi16g04000\_t001 |  | \| \| |  |  |  |  |  |  |  |  |  |  |
| 1 | Vvi-Vitvi16g00004\_t001 |  | \| \| |  |  |  |  |  |  |  |  |  |  |
| 2 | Vvi-Vitvi16g00005\_t001 |  | \| \| |  | Atr-ERN10220 |  |  |  |  |  |  |  |  |
| 2 | Vvi-Vitvi16g04001\_t001 |  | \| \| |  | \| \| |  |  |  |  |  |  |  |  |
| 2 | Vvi-Vitvi16g00007\_t001 |  | \| \| |  | \| \| |  |  |  |  |  |  |  |  |
| 2 | Vvi-Vitvi16g00008\_t001 |  | \| \| |  | \| \| |  |  |  |  |  |  |  |  |
| 2 | Vvi-Vitvi16g00010\_t001 |  | Atr-ERN10219 |  | Atr-ERN10219 |  |  |  |  |  |  |  |  |
| 2 | Vvi-Vitvi16g00011\_t001 |  | \| \| |  | Atr-ERN10218 |  |  |  |  |  |  |  |  |
| 2 | Vvi-Vitvi16g01488\_t001 |  | \| \| |  | \| \| |  |  |  |  |  |  |  |  |
| 2 | Vvi-Vitvi16g00012\_t001 |  | \| \| |  | \| \| |  |  |  |  |  |  |  |  |
| 2 | Vvi-Vitvi16g00013\_t001 |  | \| \| |  | Atr-ERN10214 |  |  |  |  |  |  |  |  |
| 2 | Vvi-Vitvi16g04002\_t001 |  | \| \| |  | Atr-ERN10213 |  |  |  |  |  |  |  |  |
| 2 | Vvi-Vitvi16g04003\_t001 |  | \| \| |  | \| \| |  |  |  |  |  |  |  |  |
| 2 | Vvi-Vitvi16g00015\_t001 |  | \| \| |  | Atr-ERN10211 |  |  |  |  |  |  |  |  |
| 1 | Vvi-Vitvi16g00016\_t001 |  | Atr-ERN10228 |  |  |  |  |  |  |  |  |  |  |
| 1 | Vvi-Vitvi16g00017\_t001 |  | Atr-ERN10232 |  |  |  |  |  |  |  |  |  |  |
| 1 | Vvi-Vitvi16g00018\_t001 |  | Atr-ERN10235 |  |  |  |  |  |  |  |  |  |  |
| 1 | Vvi-Vitvi16g01489\_t001 |  | Atr-ERN10236 |  |  |  |  |  |  |  |  |  |  |
| 1 | Vvi-Vitvi16g00019\_t001 |  | Atr-ERN10237 |  |  |  |  |  |  |  |  |  |  |
| 1 | Vvi-Vitvi16g00020\_t001 |  | Atr-ERN10240 |  |  |  |  |  |  |  |  |  |  |
| 1 | Vvi-Vitvi16g00021\_t001 |  | Atr-ERN10241 |  |  |  |  |  |  |  |  |  |  |
| 1 | Vvi-Vitvi16g00022\_t001 |  | Atr-ERN10242 |  |  |  |  |  |  |  |  |  |  |
| 0 | Vvi-Vitvi16g00024\_t002 |  |  |  |  |  |  |  |  |  |  |  |  |
| 0 | Vvi-Vitvi16g00025\_t001 |  |  |  |  |  |  |  |  |  |  |  |  |
| 0 | Vvi-Vitvi16g00026\_t001 |  |  |  |  |  |  |  |  |  |  |  |  |
| 0 | Vvi-Vitvi16g01490\_t001 |  |  |  |  |  |  |  |  |  |  |  |  |
| 0 | Vvi-Vitvi16g04004\_t001 |  |  |  |  |  |  |  |  |  |  |  |  |
| 0 | Vvi-Vitvi16g00028\_t001 |  |  |  |  |  |  |  |  |  |  |  |  |
| 0 | Vvi-Vitvi16g00030\_t001 |  |  |  |  |  |  |  |  |  |  |  |  |
| 0 | Vvi-Vitvi16g00031\_t002 |  |  |  |  |  |  |  |  |  |  |  |  |
| 0 | Vvi-Vitvi16g00032\_t001 |  |  |  |  |  |  |  |  |  |  |  |  |
| 0 | Vvi-Vitvi16g00033\_t003 |  |  |  |  |  |  |  |  |  |  |  |  |
| 0 | Vvi-Vitvi16g00034\_t001 |  |  |  |  |  |  |  |  |  |  |  |  |
| 0 | Vvi-Vitvi16g00035\_t001 |  |  |  |  |  |  |  |  |  |  |  |  |
| 0 | Vvi-Vitvi16g00036\_t001 |  |  |  |  |  |  |  |  |  |  |  |  |
| 0 | Vvi-Vitvi16g00037\_t001 |  |  |  |  |  |  |  |  |  |  |  |  |
| 0 | Vvi-Vitvi16g00038\_t001 |  |  |  |  |  |  |  |  |  |  |  |  |
| 0 | Vvi-Vitvi16g01491\_t001 |  |  |  |  |  |  |  |  |  |  |  |  |
| 0 | Vvi-Vitvi16g00040\_t001 |  |  |  |  |  |  |  |  |  |  |  |  |
| 0 | Vvi-Vitvi16g04005\_t001 |  |  |  |  |  |  |  |  |  |  |  |  |
| 0 | Vvi-Vitvi16g00041\_t001 |  |  |  |  |  |  |  |  |  |  |  |  |
| 0 | Vvi-Vitvi16g01493\_t001 |  |  |  |  |  |  |  |  |  |  |  |  |
| 0 | Vvi-Vitvi16g00042\_t001 |  |  |  |  |  |  |  |  |  |  |  |  |
| 0 | Vvi-Vitvi16g04006\_t001 |  |  |  |  |  |  |  |  |  |  |  |  |
| 0 | Vvi-Vitvi16g04007\_t001 |  |  |  |  |  |  |  |  |  |  |  |  |
| 0 | Vvi-Vitvi16g01495\_t001 |  |  |  |  |  |  |  |  |  |  |  |  |
| 0 | Vvi-Vitvi16g00043\_t001 |  |  |  |  |  |  |  |  |  |  |  |  |
| 0 | Vvi-Vitvi16g01496\_t001 |  |  |  |  |  |  |  |  |  |  |  |  |
| 0 | Vvi-Vitvi16g00044\_t001 |  |  |  |  |  |  |  |  |  |  |  |  |
| 0 | Vvi-Vitvi16g01497\_t001 |  |  |  |  |  |  |  |  |  |  |  |  |
| 0 | Vvi-Vitvi16g00045\_t001 |  |  |  |  |  |  |  |  |  |  |  |  |
| 0 | Vvi-Vitvi16g01498\_t001 |  |  |  |  |  |  |  |  |  |  |  |  |
| 0 | Vvi-Vitvi16g00046\_t001 |  |  |  |  |  |  |  |  |  |  |  |  |
| 0 | Vvi-Vitvi16g00048\_t001 |  |  |  |  |  |  |  |  |  |  |  |  |
| 0 | Vvi-Vitvi16g04008\_t001 |  |  |  |  |  |  |  |  |  |  |  |  |
| 0 | Vvi-Vitvi16g04009\_t001 |  |  |  |  |  |  |  |  |  |  |  |  |
| 0 | Vvi-Vitvi16g01500\_t001 |  |  |  |  |  |  |  |  |  |  |  |  |
| 0 | Vvi-Vitvi16g01501\_t001 |  |  |  |  |  |  |  |  |  |  |  |  |
| 0 | Vvi-Vitvi16g04010\_t001 |  |  |  |  |  |  |  |  |  |  |  |  |
| 0 | Vvi-Vitvi16g04011\_t001 |  |  |  |  |  |  |  |  |  |  |  |  |
| 0 | Vvi-Vitvi16g00053\_t001 |  |  |  |  |  |  |  |  |  |  |  |  |
| 0 | Vvi-Vitvi16g00056\_t001 |  |  |  |  |  |  |  |  |  |  |  |  |
| 0 | Vvi-Vitvi16g04012\_t001 |  |  |  |  |  |  |  |  |  |  |  |  |
| 0 | Vvi-Vitvi16g01506\_t001 |  |  |  |  |  |  |  |  |  |  |  |  |
| 0 | Vvi-Vitvi16g00062\_t001 |  |  |  |  |  |  |  |  |  |  |  |  |
| 0 | Vvi-Vitvi16g04013\_t001 |  |  |  |  |  |  |  |  |  |  |  |  |
| 0 | Vvi-Vitvi16g00065\_t001 |  |  |  |  |  |  |  |  |  |  |  |  |
| 0 | Vvi-Vitvi16g00067\_t001 |  |  |  |  |  |  |  |  |  |  |  |  |
| 0 | Vvi-Vitvi16g00068\_t001 |  |  |  |  |  |  |  |  |  |  |  |  |
| 0 | Vvi-Vitvi16g04014\_t001 |  |  |  |  |  |  |  |  |  |  |  |  |
| 0 | Vvi-Vitvi16g04015\_t001 |  |  |  |  |  |  |  |  |  |  |  |  |
| 0 | Vvi-Vitvi16g01508\_t001 |  |  |  |  |  |  |  |  |  |  |  |  |
| 0 | Vvi-Vitvi16g00069\_t001 |  |  |  |  |  |  |  |  |  |  |  |  |
| 1 | Vvi-Vitvi16g00070\_t001 |  | Atr-ERN15688 |  |  |  |  |  |  |  |  |  |  |
| 1 | Vvi-Vitvi16g01509\_t001 |  | Atr-ERN15686 |  |  |  |  |  |  |  |  |  |  |
| 1 | Vvi-Vitvi16g00071\_t004 |  | Atr-ERN15683 |  |  |  |  |  |  |  |  |  |  |
| 1 | Vvi-Vitvi16g04016\_t001 |  | Atr-ERN15681 |  |  |  |  |  |  |  |  |  |  |
| 1 | Vvi-Vitvi16g04017\_t001 |  | \| \| |  |  |  |  |  |  |  |  |  |  |
| 1 | Vvi-Vitvi16g00073\_t001 |  | Atr-ERN15677 |  |  |  |  |  |  |  |  |  |  |
| 1 | Vvi-Vitvi16g04018\_t001 |  | \| \| |  |  |  |  |  |  |  |  |  |  |
| 1 | Vvi-Vitvi16g01510\_t001 |  | \| \| |  |  |  |  |  |  |  |  |  |  |
| 1 | Vvi-Vitvi16g04019\_t001 |  | \| \| |  |  |  |  |  |  |  |  |  |  |
| 1 | Vvi-Vitvi16g00075\_t001 |  | \| \| |  |  |  |  |  |  |  |  |  |  |
| 1 | Vvi-Vitvi16g00076\_t001 |  | \| \| |  |  |  |  |  |  |  |  |  |  |
| 1 | Vvi-Vitvi16g00077\_t001 |  | \| \| |  |  |  |  |  |  |  |  |  |  |
| 1 | Vvi-Vitvi16g04020\_t001 |  | \| \| |  |  |  |  |  |  |  |  |  |  |
| 1 | Vvi-Vitvi16g00079\_t001 |  | Atr-ERN15660 |  |  |  |  |  |  |  |  |  |  |
| 0 | Vvi-Vitvi16g00080\_t004 |  |  |  |  |  |  |  |  |  |  |  |  |
| 0 | Vvi-Vitvi16g01511\_t001 |  |  |  |  |  |  |  |  |  |  |  |  |
| 0 | Vvi-Vitvi16g00082\_t001 |  |  |  |  |  |  |  |  |  |  |  |  |
| 0 | Vvi-Vitvi16g01512\_t001 |  |  |  |  |  |  |  |  |  |  |  |  |
| 0 | Vvi-Vitvi16g00083\_t001 |  |  |  |  |  |  |  |  |  |  |  |  |
| 0 | Vvi-Vitvi16g00084\_t001 |  |  |  |  |  |  |  |  |  |  |  |  |
| 0 | Vvi-Vitvi16g00086\_t001 |  |  |  |  |  |  |  |  |  |  |  |  |
| 0 | Vvi-Vitvi16g00087\_t001 |  |  |  |  |  |  |  |  |  |  |  |  |
| 0 | Vvi-Vitvi16g00088\_t001 |  |  |  |  |  |  |  |  |  |  |  |  |
| 0 | Vvi-Vitvi16g01513\_t001 |  |  |  |  |  |  |  |  |  |  |  |  |
| 0 | Vvi-Vitvi16g00089\_t001 |  |  |  |  |  |  |  |  |  |  |  |  |
| 0 | Vvi-Vitvi16g04021\_t001 |  |  |  |  |  |  |  |  |  |  |  |  |
| 0 | Vvi-Vitvi16g04022\_t001 |  |  |  |  |  |  |  |  |  |  |  |  |
| 0 | Vvi-Vitvi16g00093\_t003 |  |  |  |  |  |  |  |  |  |  |  |  |
| 0 | Vvi-Vitvi16g00094\_t001 |  |  |  |  |  |  |  |  |  |  |  |  |
| 0 | Vvi-Vitvi16g00095\_t001 |  |  |  |  |  |  |  |  |  |  |  |  |
| 0 | Vvi-Vitvi16g01514\_t001 |  |  |  |  |  |  |  |  |  |  |  |  |
| 0 | Vvi-Vitvi16g00096\_t001 |  |  |  |  |  |  |  |  |  |  |  |  |
| 0 | Vvi-Vitvi16g00097\_t001 |  |  |  |  |  |  |  |  |  |  |  |  |
| 0 | Vvi-Vitvi16g00098\_t001 |  |  |  |  |  |  |  |  |  |  |  |  |
| 0 | Vvi-Vitvi16g04023\_t001 |  |  |  |  |  |  |  |  |  |  |  |  |
| 0 | Vvi-Vitvi16g01448\_t001 |  |  |  |  |  |  |  |  |  |  |  |  |
| 0 | Vvi-Vitvi16g00102\_t001 |  |  |  |  |  |  |  |  |  |  |  |  |
| 0 | Vvi-Vitvi16g04024\_t001 |  |  |  |  |  |  |  |  |  |  |  |  |
| 0 | Vvi-Vitvi16g04025\_t001 |  |  |  |  |  |  |  |  |  |  |  |  |
| 0 | Vvi-Vitvi16g04026\_t001 |  |  |  |  |  |  |  |  |  |  |  |  |
| 0 | Vvi-Vitvi16g04027\_t001 |  |  |  |  |  |  |  |  |  |  |  |  |
| 0 | Vvi-Vitvi16g04028\_t001 |  |  |  |  |  |  |  |  |  |  |  |  |
| 0 | Vvi-Vitvi16g04029\_t001 |  |  |  |  |  |  |  |  |  |  |  |  |
| 0 | Vvi-Vitvi16g04030\_t001 |  |  |  |  |  |  |  |  |  |  |  |  |
| 0 | Vvi-Vitvi16g01515\_t001 |  |  |  |  |  |  |  |  |  |  |  |  |
| 0 | Vvi-Vitvi16g04031\_t001 |  |  |  |  |  |  |  |  |  |  |  |  |
| 0 | Vvi-Vitvi16g00106\_t001 |  |  |  |  |  |  |  |  |  |  |  |  |
| 0 | Vvi-Vitvi16g04032\_t001 |  |  |  |  |  |  |  |  |  |  |  |  |
| 0 | Vvi-Vitvi16g04033\_t001 |  |  |  |  |  |  |  |  |  |  |  |  |
| 0 | Vvi-Vitvi16g04034\_t001 |  |  |  |  |  |  |  |  |  |  |  |  |
| 0 | Vvi-Vitvi16g04035\_t001 |  |  |  |  |  |  |  |  |  |  |  |  |
| 0 | Vvi-Vitvi16g04036\_t001 |  |  |  |  |  |  |  |  |  |  |  |  |
| 0 | Vvi-Vitvi16g04037\_t001 |  |  |  |  |  |  |  |  |  |  |  |  |
| 0 | Vvi-Vitvi16g01521\_t001 |  |  |  |  |  |  |  |  |  |  |  |  |
| 0 | Vvi-Vitvi16g01522\_t001 |  |  |  |  |  |  |  |  |  |  |  |  |
| 0 | Vvi-Vitvi16g04038\_t001 |  |  |  |  |  |  |  |  |  |  |  |  |
| 0 | Vvi-Vitvi16g01523\_t001 |  |  |  |  |  |  |  |  |  |  |  |  |
| 0 | Vvi-Vitvi16g01524\_t001 |  |  |  |  |  |  |  |  |  |  |  |  |
| 0 | Vvi-Vitvi16g04039\_t001 |  |  |  |  |  |  |  |  |  |  |  |  |
| 0 | Vvi-Vitvi16g04040\_t001 |  |  |  |  |  |  |  |  |  |  |  |  |
| 0 | Vvi-Vitvi16g01525\_t001 |  |  |  |  |  |  |  |  |  |  |  |  |
| 1 | Vvi-Vitvi16g01526\_t001 |  | Atr-ERN15493 |  |  |  |  |  |  |  |  |  |  |
| 1 | Vvi-Vitvi16g00109\_t001 |  | \| \| |  |  |  |  |  |  |  |  |  |  |
| 1 | Vvi-Vitvi16g01527\_t001 |  | \| \| |  |  |  |  |  |  |  |  |  |  |
| 1 | Vvi-Vitvi16g01528\_t001 |  | \| \| |  |  |  |  |  |  |  |  |  |  |
| 1 | Vvi-Vitvi16g00110\_t001 |  | \| \| |  |  |  |  |  |  |  |  |  |  |
| 1 | Vvi-Vitvi16g00111\_t001 |  | Atr-ERN15511 |  |  |  |  |  |  |  |  |  |  |
| 1 | Vvi-Vitvi16g00112\_t001 |  | Atr-ERN15514 |  |  |  |  |  |  |  |  |  |  |
| 1 | Vvi-Vitvi16g01529\_t001 |  | \| \| |  |  |  |  |  |  |  |  |  |  |
| 1 | Vvi-Vitvi16g00114\_t001 |  | Atr-ERN15515 |  |  |  |  |  |  |  |  |  |  |
| 1 | Vvi-Vitvi16g00115\_t001 |  | \| \| |  |  |  |  |  |  |  |  |  |  |
| 1 | Vvi-Vitvi16g00116\_t001 |  | Atr-ERN15517 |  |  |  |  |  |  |  |  |  |  |
| 1 | Vvi-Vitvi16g00118\_t001 |  | \| \| |  |  |  |  |  |  |  |  |  |  |
| 1 | Vvi-Vitvi16g00119\_t001 |  | Atr-ERN15520 |  |  |  |  |  |  |  |  |  |  |
| 1 | Vvi-Vitvi16g00120\_t001 |  | Atr-ERN15528 |  |  |  |  |  |  |  |  |  |  |
| 1 | Vvi-Vitvi16g00121\_t001 |  | \| \| |  |  |  |  |  |  |  |  |  |  |
| 1 | Vvi-Vitvi16g00122\_t001 |  | \| \| |  |  |  |  |  |  |  |  |  |  |
| 1 | Vvi-Vitvi16g01449\_t002 |  | \| \| |  |  |  |  |  |  |  |  |  |  |
| 1 | Vvi-Vitvi16g00123\_t001 |  | \| \| |  |  |  |  |  |  |  |  |  |  |
| 1 | Vvi-Vitvi16g00125\_t001 |  | \| \| |  |  |  |  |  |  |  |  |  |  |
| 1 | Vvi-Vitvi16g04041\_t001 |  | \| \| |  |  |  |  |  |  |  |  |  |  |
| 1 | Vvi-Vitvi16g00126\_t001 |  | \| \| |  |  |  |  |  |  |  |  |  |  |
| 1 | Vvi-Vitvi16g00129\_t001 |  | \| \| |  |  |  |  |  |  |  |  |  |  |
| 1 | Vvi-Vitvi16g04042\_t001 |  | \| \| |  |  |  |  |  |  |  |  |  |  |
| 1 | Vvi-Vitvi16g04043\_t001 |  | \| \| |  |  |  |  |  |  |  |  |  |  |
| 1 | Vvi-Vitvi16g00133\_t001 |  | \| \| |  |  |  |  |  |  |  |  |  |  |
| 1 | Vvi-Vitvi16g04044\_t001 |  | \| \| |  |  |  |  |  |  |  |  |  |  |
| 1 | Vvi-Vitvi16g00135\_t002 |  | \| \| |  |  |  |  |  |  |  |  |  |  |
| 1 | Vvi-Vitvi16g04045\_t001 |  | \| \| |  |  |  |  |  |  |  |  |  |  |
| 1 | Vvi-Vitvi16g00136\_t001 |  | Atr-ERN15542 |  |  |  |  |  |  |  |  |  |  |
| 1 | Vvi-Vitvi16g01534\_t001 |  | \| \| |  |  |  |  |  |  |  |  |  |  |
| 1 | Vvi-Vitvi16g00137\_t001 |  | \| \| |  |  |  |  |  |  |  |  |  |  |
| 1 | Vvi-Vitvi16g04046\_t001 |  | \| \| |  |  |  |  |  |  |  |  |  |  |
| 1 | Vvi-Vitvi16g00138\_t001 |  | \| \| |  |  |  |  |  |  |  |  |  |  |
| 1 | Vvi-Vitvi16g00139\_t001 |  | \| \| |  |  |  |  |  |  |  |  |  |  |
| 1 | Vvi-Vitvi16g00142\_t001 |  | \| \| |  |  |  |  |  |  |  |  |  |  |
| 1 | Vvi-Vitvi16g04047\_t001 |  | \| \| |  |  |  |  |  |  |  |  |  |  |
| 1 | Vvi-Vitvi16g00144\_t001 |  | \| \| |  |  |  |  |  |  |  |  |  |  |
| 1 | Vvi-Vitvi16g00145\_t001 |  | \| \| |  |  |  |  |  |  |  |  |  |  |
| 1 | Vvi-Vitvi16g00146\_t001 |  | \| \| |  |  |  |  |  |  |  |  |  |  |
| 1 | Vvi-Vitvi16g00147\_t001 |  | Atr-ERN15560 |  |  |  |  |  |  |  |  |  |  |
| 1 | Vvi-Vitvi16g00148\_t001 |  | \| \| |  |  |  |  |  |  |  |  |  |  |
| 1 | Vvi-Vitvi16g00149\_t001 |  | Atr-ERN15568 |  |  |  |  |  |  |  |  |  |  |
| 1 | Vvi-Vitvi16g01536\_t001 |  | \| \| |  |  |  |  |  |  |  |  |  |  |
| 1 | Vvi-Vitvi16g00150\_t001 |  | Atr-ERN15574 |  |  |  |  |  |  |  |  |  |  |
| 1 | Vvi-Vitvi16g01537\_t001 |  | Atr-ERN15576 |  |  |  |  |  |  |  |  |  |  |
| 1 | Vvi-Vitvi16g01538\_t001 |  | Atr-ERN15578 |  |  |  |  |  |  |  |  |  |  |
| 1 | Vvi-Vitvi16g00151\_t001 |  | Atr-ERN15581 |  |  |  |  |  |  |  |  |  |  |
| 1 | Vvi-Vitvi16g04048\_t001 |  | \| \| |  |  |  |  |  |  |  |  |  |  |
| 1 | Vvi-Vitvi16g00152\_t001 |  | \| \| |  |  |  |  |  |  |  |  |  |  |
| 1 | Vvi-Vitvi16g04049\_t001 |  | \| \| |  |  |  |  |  |  |  |  |  |  |
| 1 | Vvi-Vitvi16g04050\_t001 |  | \| \| |  |  |  |  |  |  |  |  |  |  |
| 1 | Vvi-Vitvi16g00153\_t001 |  | \| \| |  |  |  |  |  |  |  |  |  |  |
| 1 | Vvi-Vitvi16g00154\_t001 |  | Atr-ERN15584 |  |  |  |  |  |  |  |  |  |  |
| 1 | Vvi-Vitvi16g00155\_t001 |  | \| \| |  |  |  |  |  |  |  |  |  |  |
| 1 | Vvi-Vitvi16g00156\_t001 |  | \| \| |  |  |  |  |  |  |  |  |  |  |
| 1 | Vvi-Vitvi16g01539\_t001 |  | Atr-ERN15591 |  |  |  |  |  |  |  |  |  |  |
| 1 | Vvi-Vitvi16g00158\_t001 |  | \| \| |  |  |  |  |  |  |  |  |  |  |
| 1 | Vvi-Vitvi16g00159\_t001 |  | Atr-ERN15592 |  |  |  |  |  |  |  |  |  |  |
| 1 | Vvi-Vitvi16g01540\_t003 |  | \| \| |  |  |  |  |  |  |  |  |  |  |
| 1 | Vvi-Vitvi16g01542\_t001 |  | \| \| |  |  |  |  |  |  |  |  |  |  |
| 1 | Vvi-Vitvi16g00160\_t001 |  | \| \| |  |  |  |  |  |  |  |  |  |  |
| 1 | Vvi-Vitvi16g04051\_t001 |  | \| \| |  |  |  |  |  |  |  |  |  |  |
| 1 | Vvi-Vitvi16g04052\_t001 |  | \| \| |  |  |  |  |  |  |  |  |  |  |
| 1 | Vvi-Vitvi16g01544\_t001 |  | \| \| |  |  |  |  |  |  |  |  |  |  |
| 1 | Vvi-Vitvi16g01545\_t001 |  | \| \| |  |  |  |  |  |  |  |  |  |  |
| 1 | Vvi-Vitvi16g01546\_t001 |  | \| \| |  |  |  |  |  |  |  |  |  |  |
| 1 | Vvi-Vitvi16g04053\_t001 |  | \| \| |  |  |  |  |  |  |  |  |  |  |
| 1 | Vvi-Vitvi16g04054\_t001 |  | \| \| |  |  |  |  |  |  |  |  |  |  |
| 1 | Vvi-Vitvi16g04055\_t001 |  | \| \| |  |  |  |  |  |  |  |  |  |  |
| 1 | Vvi-Vitvi16g01549\_t001 |  | \| \| |  |  |  |  |  |  |  |  |  |  |
| 1 | Vvi-Vitvi16g00163\_t001 |  | \| \| |  |  |  |  |  |  |  |  |  |  |
| 1 | Vvi-Vitvi16g04056\_t001 |  | \| \| |  |  |  |  |  |  |  |  |  |  |
| 1 | Vvi-Vitvi16g00165\_t001 |  | \| \| |  |  |  |  |  |  |  |  |  |  |
| 1 | Vvi-Vitvi16g04057\_t001 |  | \| \| |  |  |  |  |  |  |  |  |  |  |
| 1 | Vvi-Vitvi16g04058\_t001 |  | \| \| |  |  |  |  |  |  |  |  |  |  |
| 1 | Vvi-Vitvi16g00168\_t002 |  | Atr-ERN15594 |  |  |  |  |  |  |  |  |  |  |
| 1 | Vvi-Vitvi16g04059\_t001 |  | \| \| |  |  |  |  |  |  |  |  |  |  |
| 1 | Vvi-Vitvi16g00170\_t001 |  | \| \| |  |  |  |  |  |  |  |  |  |  |
| 1 | Vvi-Vitvi16g00173\_t001 |  | \| \| |  |  |  |  |  |  |  |  |  |  |
| 1 | Vvi-Vitvi16g00174\_t001 |  | Atr-ERN15616 |  |  |  |  |  |  |  |  |  |  |
| 1 | Vvi-Vitvi16g04060\_t001 |  | \| \| |  |  |  |  |  |  |  |  |  |  |
| 1 | Vvi-Vitvi16g01550\_t001 |  | \| \| |  |  |  |  |  |  |  |  |  |  |
| 1 | Vvi-Vitvi16g04061\_t001 |  | \| \| |  |  |  |  |  |  |  |  |  |  |
| 1 | Vvi-Vitvi16g04062\_t001 |  | \| \| |  |  |  |  |  |  |  |  |  |  |
| 1 | Vvi-Vitvi16g00177\_t001 |  | Atr-ERN15625 |  |  |  |  |  |  |  |  |  |  |
| 1 | Vvi-Vitvi16g00178\_t001 |  | Atr-ERN15627 |  |  |  |  |  |  |  |  |  |  |
| 1 | Vvi-Vitvi16g00179\_t001 |  | Atr-ERN15628 |  |  |  |  |  |  |  |  |  |  |
| 1 | Vvi-Vitvi16g00180\_t001 |  | Atr-ERN15629 |  |  |  |  |  |  |  |  |  |  |
| 1 | Vvi-Vitvi16g00181\_t001 |  | Atr-ERN15633 |  |  |  |  |  |  |  |  |  |  |
| 0 | Vvi-Vitvi16g00182\_t001 |  |  |  |  |  |  |  |  |  |  |  |  |
| 0 | Vvi-Vitvi16g00183\_t001 |  |  |  |  |  |  |  |  |  |  |  |  |
| 0 | Vvi-Vitvi16g00184\_t001 |  |  |  |  |  |  |  |  |  |  |  |  |
| 0 | Vvi-Vitvi16g04063\_t001 |  |  |  |  |  |  |  |  |  |  |  |  |
| 0 | Vvi-Vitvi16g01552\_t001 |  |  |  |  |  |  |  |  |  |  |  |  |
| 0 | Vvi-Vitvi16g04064\_t001 |  |  |  |  |  |  |  |  |  |  |  |  |
| 0 | Vvi-Vitvi16g04065\_t001 |  |  |  |  |  |  |  |  |  |  |  |  |
| 0 | Vvi-Vitvi16g04066\_t001 |  |  |  |  |  |  |  |  |  |  |  |  |
| 0 | Vvi-Vitvi16g00188\_t001 |  |  |  |  |  |  |  |  |  |  |  |  |
| 0 | Vvi-Vitvi16g01555\_t001 |  |  |  |  |  |  |  |  |  |  |  |  |
| 0 | Vvi-Vitvi16g00189\_t001 |  |  |  |  |  |  |  |  |  |  |  |  |
| 0 | Vvi-Vitvi16g00190\_t001 |  |  |  |  |  |  |  |  |  |  |  |  |
| 0 | Vvi-Vitvi16g01556\_t001 |  |  |  |  |  |  |  |  |  |  |  |  |
| 0 | Vvi-Vitvi16g04067\_t001 |  |  |  |  |  |  |  |  |  |  |  |  |
| 0 | Vvi-Vitvi16g00194\_t002 |  |  |  |  |  |  |  |  |  |  |  |  |
| 0 | Vvi-Vitvi16g00195\_t001 |  |  |  |  |  |  |  |  |  |  |  |  |
| 0 | Vvi-Vitvi16g04068\_t001 |  |  |  |  |  |  |  |  |  |  |  |  |
| 0 | Vvi-Vitvi16g04069\_t001 |  |  |  |  |  |  |  |  |  |  |  |  |
| 0 | Vvi-Vitvi16g04070\_t001 |  |  |  |  |  |  |  |  |  |  |  |  |
| 0 | Vvi-Vitvi16g00196\_t001 |  |  |  |  |  |  |  |  |  |  |  |  |
| 0 | Vvi-Vitvi16g00197\_t001 |  |  |  |  |  |  |  |  |  |  |  |  |
| 0 | Vvi-Vitvi16g01559\_t001 |  |  |  |  |  |  |  |  |  |  |  |  |
| 0 | Vvi-Vitvi16g04071\_t001 |  |  |  |  |  |  |  |  |  |  |  |  |
| 0 | Vvi-Vitvi16g04072\_t001 |  |  |  |  |  |  |  |  |  |  |  |  |
| 0 | Vvi-Vitvi16g01561\_t001 |  |  |  |  |  |  |  |  |  |  |  |  |
| 0 | Vvi-Vitvi16g00199\_t001 |  |  |  |  |  |  |  |  |  |  |  |  |
| 0 | Vvi-Vitvi16g01562\_t001 |  |  |  |  |  |  |  |  |  |  |  |  |
| 0 | Vvi-Vitvi16g04073\_t001 |  |  |  |  |  |  |  |  |  |  |  |  |
| 0 | Vvi-Vitvi16g04074\_t001 |  |  |  |  |  |  |  |  |  |  |  |  |
| 0 | Vvi-Vitvi16g01564\_t001 |  |  |  |  |  |  |  |  |  |  |  |  |
| 0 | Vvi-Vitvi16g04075\_t001 |  |  |  |  |  |  |  |  |  |  |  |  |
| 0 | Vvi-Vitvi16g04076\_t001 |  |  |  |  |  |  |  |  |  |  |  |  |
| 0 | Vvi-Vitvi16g04077\_t001 |  |  |  |  |  |  |  |  |  |  |  |  |
| 0 | Vvi-Vitvi16g04078\_t001 |  |  |  |  |  |  |  |  |  |  |  |  |
| 0 | Vvi-Vitvi16g00202\_t001 |  |  |  |  |  |  |  |  |  |  |  |  |
| 0 | Vvi-Vitvi16g00203\_t001 |  |  |  |  |  |  |  |  |  |  |  |  |
| 0 | Vvi-Vitvi16g00204\_t001 |  |  |  |  |  |  |  |  |  |  |  |  |
| 0 | Vvi-Vitvi16g01566\_t001 |  |  |  |  |  |  |  |  |  |  |  |  |
| 0 | Vvi-Vitvi16g00205\_t001 |  |  |  |  |  |  |  |  |  |  |  |  |
| 0 | Vvi-Vitvi16g04079\_t001 |  |  |  |  |  |  |  |  |  |  |  |  |
| 0 | Vvi-Vitvi16g04080\_t001 |  |  |  |  |  |  |  |  |  |  |  |  |
| 0 | Vvi-Vitvi16g01568\_t001 |  |  |  |  |  |  |  |  |  |  |  |  |
| 0 | Vvi-Vitvi16g01569\_t001 |  |  |  |  |  |  |  |  |  |  |  |  |
| 0 | Vvi-Vitvi16g01572\_t001 |  |  |  |  |  |  |  |  |  |  |  |  |
| 0 | Vvi-Vitvi16g00211\_t001 |  |  |  |  |  |  |  |  |  |  |  |  |
| 0 | Vvi-Vitvi16g04081\_t001 |  |  |  |  |  |  |  |  |  |  |  |  |
| 0 | Vvi-Vitvi16g04082\_t001 |  |  |  |  |  |  |  |  |  |  |  |  |
| 0 | Vvi-Vitvi16g04083\_t001 |  |  |  |  |  |  |  |  |  |  |  |  |
| 0 | Vvi-Vitvi16g04084\_t001 |  |  |  |  |  |  |  |  |  |  |  |  |
| 0 | Vvi-Vitvi16g04085\_t001 |  |  |  |  |  |  |  |  |  |  |  |  |
| 0 | Vvi-Vitvi16g04086\_t001 |  |  |  |  |  |  |  |  |  |  |  |  |
| 0 | Vvi-Vitvi16g01616\_t001 |  |  |  |  |  |  |  |  |  |  |  |  |
| 0 | Vvi-Vitvi16g01618\_t001 |  |  |  |  |  |  |  |  |  |  |  |  |
| 0 | Vvi-Vitvi16g04087\_t001 |  |  |  |  |  |  |  |  |  |  |  |  |
| 0 | Vvi-Vitvi16g00256\_t001 |  |  |  |  |  |  |  |  |  |  |  |  |
| 0 | Vvi-Vitvi16g04088\_t001 |  |  |  |  |  |  |  |  |  |  |  |  |
| 0 | Vvi-Vitvi16g04089\_t001 |  |  |  |  |  |  |  |  |  |  |  |  |
| 0 | Vvi-Vitvi16g00258\_t001 |  |  |  |  |  |  |  |  |  |  |  |  |
| 0 | Vvi-Vitvi16g04090\_t001 |  |  |  |  |  |  |  |  |  |  |  |  |
| 0 | Vvi-Vitvi16g01623\_t001 |  |  |  |  |  |  |  |  |  |  |  |  |
| 0 | Vvi-Vitvi16g00264\_t001 |  |  |  |  |  |  |  |  |  |  |  |  |
| 0 | Vvi-Vitvi16g00265\_t001 |  |  |  |  |  |  |  |  |  |  |  |  |
| 0 | Vvi-Vitvi16g04091\_t001 |  |  |  |  |  |  |  |  |  |  |  |  |
| 0 | Vvi-Vitvi16g04092\_t001 |  |  |  |  |  |  |  |  |  |  |  |  |
| 0 | Vvi-Vitvi16g01626\_t001 |  |  |  |  |  |  |  |  |  |  |  |  |
| 0 | Vvi-Vitvi16g01627\_t001 |  |  |  |  |  |  |  |  |  |  |  |  |
| 0 | Vvi-Vitvi16g00268\_t001 |  |  |  |  |  |  |  |  |  |  |  |  |
| 0 | Vvi-Vitvi16g01628\_t001 |  |  |  |  |  |  |  |  |  |  |  |  |
| 0 | Vvi-Vitvi16g04093\_t001 |  |  |  |  |  |  |  |  |  |  |  |  |
| 0 | Vvi-Vitvi16g04094\_t001 |  |  |  |  |  |  |  |  |  |  |  |  |
| 0 | Vvi-Vitvi16g00271\_t001 |  |  |  |  |  |  |  |  |  |  |  |  |
| 0 | Vvi-Vitvi16g01629\_t001 |  |  |  |  |  |  |  |  |  |  |  |  |
| 0 | Vvi-Vitvi16g00272\_t001 |  |  |  |  |  |  |  |  |  |  |  |  |
| 0 | Vvi-Vitvi16g00273\_t001 |  |  |  |  |  |  |  |  |  |  |  |  |
| 0 | Vvi-Vitvi16g04095\_t001 |  |  |  |  |  |  |  |  |  |  |  |  |
| 0 | Vvi-Vitvi16g00274\_t001 |  |  |  |  |  |  |  |  |  |  |  |  |
| 0 | Vvi-Vitvi16g00275\_t001 |  |  |  |  |  |  |  |  |  |  |  |  |
| 0 | Vvi-Vitvi16g00276\_t001 |  |  |  |  |  |  |  |  |  |  |  |  |
| 0 | Vvi-Vitvi16g04096\_t001 |  |  |  |  |  |  |  |  |  |  |  |  |
| 0 | Vvi-Vitvi16g00278\_t001 |  |  |  |  |  |  |  |  |  |  |  |  |
| 0 | Vvi-Vitvi16g04097\_t001 |  |  |  |  |  |  |  |  |  |  |  |  |
| 0 | Vvi-Vitvi16g04098\_t001 |  |  |  |  |  |  |  |  |  |  |  |  |
| 0 | Vvi-Vitvi16g00280\_t001 |  |  |  |  |  |  |  |  |  |  |  |  |
| 0 | Vvi-Vitvi16g04099\_t001 |  |  |  |  |  |  |  |  |  |  |  |  |
| 0 | Vvi-Vitvi16g00282\_t001 |  |  |  |  |  |  |  |  |  |  |  |  |
| 0 | Vvi-Vitvi16g04100\_t001 |  |  |  |  |  |  |  |  |  |  |  |  |
| 0 | Vvi-Vitvi16g00284\_t001 |  |  |  |  |  |  |  |  |  |  |  |  |
| 0 | Vvi-Vitvi16g00287\_t001 |  |  |  |  |  |  |  |  |  |  |  |  |
| 0 | Vvi-Vitvi16g00290\_t001 |  |  |  |  |  |  |  |  |  |  |  |  |
| 0 | Vvi-Vitvi16g01633\_t001 |  |  |  |  |  |  |  |  |  |  |  |  |
| 0 | Vvi-Vitvi16g00291\_t001 |  |  |  |  |  |  |  |  |  |  |  |  |
| 0 | Vvi-Vitvi16g04101\_t001 |  |  |  |  |  |  |  |  |  |  |  |  |
| 0 | Vvi-Vitvi16g00293\_t001 |  |  |  |  |  |  |  |  |  |  |  |  |
| 0 | Vvi-Vitvi16g04102\_t001 |  |  |  |  |  |  |  |  |  |  |  |  |
| 0 | Vvi-Vitvi16g00297\_t002 |  |  |  |  |  |  |  |  |  |  |  |  |
| 0 | Vvi-Vitvi16g00298\_t001 |  |  |  |  |  |  |  |  |  |  |  |  |
| 0 | Vvi-Vitvi16g04103\_t001 |  |  |  |  |  |  |  |  |  |  |  |  |
| 0 | Vvi-Vitvi16g01640\_t001 |  |  |  |  |  |  |  |  |  |  |  |  |
| 0 | Vvi-Vitvi16g04104\_t001 |  |  |  |  |  |  |  |  |  |  |  |  |
| 0 | Vvi-Vitvi16g04105\_t001 |  |  |  |  |  |  |  |  |  |  |  |  |
| 0 | Vvi-Vitvi16g04106\_t001 |  |  |  |  |  |  |  |  |  |  |  |  |
| 0 | Vvi-Vitvi16g01643\_t001 |  |  |  |  |  |  |  |  |  |  |  |  |
| 0 | Vvi-Vitvi16g04107\_t001 |  |  |  |  |  |  |  |  |  |  |  |  |
| 0 | Vvi-Vitvi16g00299\_t001 |  |  |  |  |  |  |  |  |  |  |  |  |
| 0 | Vvi-Vitvi16g00301\_t001 |  |  |  |  |  |  |  |  |  |  |  |  |
| 0 | Vvi-Vitvi16g00302\_t001 |  |  |  |  |  |  |  |  |  |  |  |  |
| 0 | Vvi-Vitvi16g00303\_t001 |  |  |  |  |  |  |  |  |  |  |  |  |
| 0 | Vvi-Vitvi16g00305\_t001 |  |  |  |  |  |  |  |  |  |  |  |  |
| 0 | Vvi-Vitvi16g00307\_t001 |  |  |  |  |  |  |  |  |  |  |  |  |
| 0 | Vvi-Vitvi16g04108\_t001 |  |  |  |  |  |  |  |  |  |  |  |  |
| 0 | Vvi-Vitvi16g04109\_t001 |  |  |  |  |  |  |  |  |  |  |  |  |
| 0 | Vvi-Vitvi16g04110\_t001 |  |  |  |  |  |  |  |  |  |  |  |  |
| 0 | Vvi-Vitvi16g00309\_t001 |  |  |  |  |  |  |  |  |  |  |  |  |
| 0 | Vvi-Vitvi16g04111\_t001 |  |  |  |  |  |  |  |  |  |  |  |  |
| 0 | Vvi-Vitvi16g00311\_t001 |  |  |  |  |  |  |  |  |  |  |  |  |
| 0 | Vvi-Vitvi16g00313\_t001 |  |  |  |  |  |  |  |  |  |  |  |  |
| 0 | Vvi-Vitvi16g00314\_t001 |  |  |  |  |  |  |  |  |  |  |  |  |
| 0 | Vvi-Vitvi16g04112\_t001 |  |  |  |  |  |  |  |  |  |  |  |  |
| 0 | Vvi-Vitvi16g04113\_t001 |  |  |  |  |  |  |  |  |  |  |  |  |
| 0 | Vvi-Vitvi16g04114\_t001 |  |  |  |  |  |  |  |  |  |  |  |  |
| 0 | Vvi-Vitvi16g04115\_t001 |  |  |  |  |  |  |  |  |  |  |  |  |
| 0 | Vvi-Vitvi16g04116\_t001 |  |  |  |  |  |  |  |  |  |  |  |  |
| 0 | Vvi-Vitvi16g01655\_t001 |  |  |  |  |  |  |  |  |  |  |  |  |
| 0 | Vvi-Vitvi16g00323\_t001 |  |  |  |  |  |  |  |  |  |  |  |  |
| 0 | Vvi-Vitvi16g04117\_t001 |  |  |  |  |  |  |  |  |  |  |  |  |
| 0 | Vvi-Vitvi16g00325\_t001 |  |  |  |  |  |  |  |  |  |  |  |  |
| 0 | Vvi-Vitvi16g04118\_t001 |  |  |  |  |  |  |  |  |  |  |  |  |
| 0 | Vvi-Vitvi16g00328\_t001 |  |  |  |  |  |  |  |  |  |  |  |  |
| 0 | Vvi-Vitvi16g01656\_t001 |  |  |  |  |  |  |  |  |  |  |  |  |
| 0 | Vvi-Vitvi16g00329\_t001 |  |  |  |  |  |  |  |  |  |  |  |  |
| 0 | Vvi-Vitvi16g04119\_t001 |  |  |  |  |  |  |  |  |  |  |  |  |
| 0 | Vvi-Vitvi16g04120\_t001 |  |  |  |  |  |  |  |  |  |  |  |  |
| 0 | Vvi-Vitvi16g04121\_t001 |  |  |  |  |  |  |  |  |  |  |  |  |
| 0 | Vvi-Vitvi16g01657\_t001 |  |  |  |  |  |  |  |  |  |  |  |  |
| 0 | Vvi-Vitvi16g04122\_t001 |  |  |  |  |  |  |  |  |  |  |  |  |
| 0 | Vvi-Vitvi16g00332\_t001 |  |  |  |  |  |  |  |  |  |  |  |  |
| 0 | Vvi-Vitvi16g04123\_t001 |  |  |  |  |  |  |  |  |  |  |  |  |
| 0 | Vvi-Vitvi16g04124\_t001 |  |  |  |  |  |  |  |  |  |  |  |  |
| 0 | Vvi-Vitvi16g04125\_t001 |  |  |  |  |  |  |  |  |  |  |  |  |
| 0 | Vvi-Vitvi16g04126\_t001 |  |  |  |  |  |  |  |  |  |  |  |  |
| 0 | Vvi-Vitvi16g00340\_t001 |  |  |  |  |  |  |  |  |  |  |  |  |
| 0 | Vvi-Vitvi16g00341\_t001 |  |  |  |  |  |  |  |  |  |  |  |  |
| 0 | Vvi-Vitvi16g04127\_t001 |  |  |  |  |  |  |  |  |  |  |  |  |
| 0 | Vvi-Vitvi16g04128\_t001 |  |  |  |  |  |  |  |  |  |  |  |  |
| 0 | Vvi-Vitvi16g00342\_t001 |  |  |  |  |  |  |  |  |  |  |  |  |
| 0 | Vvi-Vitvi16g04129\_t001 |  |  |  |  |  |  |  |  |  |  |  |  |
| 0 | Vvi-Vitvi16g00346\_t001 |  |  |  |  |  |  |  |  |  |  |  |  |
| 0 | Vvi-Vitvi16g00347\_t001 |  |  |  |  |  |  |  |  |  |  |  |  |
| 0 | Vvi-Vitvi16g00349\_t001 |  |  |  |  |  |  |  |  |  |  |  |  |
| 0 | Vvi-Vitvi16g00350\_t001 |  |  |  |  |  |  |  |  |  |  |  |  |
| 0 | Vvi-Vitvi16g04130\_t001 |  |  |  |  |  |  |  |  |  |  |  |  |
| 0 | Vvi-Vitvi16g00351\_t001 |  |  |  |  |  |  |  |  |  |  |  |  |
| 0 | Vvi-Vitvi16g01665\_t001 |  |  |  |  |  |  |  |  |  |  |  |  |
| 0 | Vvi-Vitvi16g01429\_t001 |  |  |  |  |  |  |  |  |  |  |  |  |
| 0 | Vvi-Vitvi16g04131\_t001 |  |  |  |  |  |  |  |  |  |  |  |  |
| 0 | Vvi-Vitvi16g01430\_t001 |  |  |  |  |  |  |  |  |  |  |  |  |
| 0 | Vvi-Vitvi16g01424\_t001 |  |  |  |  |  |  |  |  |  |  |  |  |
| 0 | Vvi-Vitvi16g01423\_t001 |  |  |  |  |  |  |  |  |  |  |  |  |
| 0 | Vvi-Vitvi16g04132\_t001 |  |  |  |  |  |  |  |  |  |  |  |  |
| 0 | Vvi-Vitvi16g01432\_t001 |  |  |  |  |  |  |  |  |  |  |  |  |
| 0 | Vvi-Vitvi16g04133\_t001 |  |  |  |  |  |  |  |  |  |  |  |  |
| 0 | Vvi-Vitvi16g04134\_t001 |  |  |  |  |  |  |  |  |  |  |  |  |
| 0 | Vvi-Vitvi16g04135\_t001 |  |  |  |  |  |  |  |  |  |  |  |  |
| 0 | Vvi-Vitvi16g04136\_t001 |  |  |  |  |  |  |  |  |  |  |  |  |
| 0 | Vvi-Vitvi16g00362\_t001 |  |  |  |  |  |  |  |  |  |  |  |  |
| 0 | Vvi-Vitvi16g00363\_t001 |  |  |  |  |  |  |  |  |  |  |  |  |
| 0 | Vvi-Vitvi16g01442\_t001 |  |  |  |  |  |  |  |  |  |  |  |  |
| 0 | Vvi-Vitvi16g00366\_t001 |  |  |  |  |  |  |  |  |  |  |  |  |
| 0 | Vvi-Vitvi16g00367\_t001 |  |  |  |  |  |  |  |  |  |  |  |  |
| 0 | Vvi-Vitvi16g00369\_t001 |  |  |  |  |  |  |  |  |  |  |  |  |
| 0 | Vvi-Vitvi16g00370\_t001 |  |  |  |  |  |  |  |  |  |  |  |  |
| 0 | Vvi-Vitvi16g00372\_t001 |  |  |  |  |  |  |  |  |  |  |  |  |
| 0 | Vvi-Vitvi16g00374\_t001 |  |  |  |  |  |  |  |  |  |  |  |  |
| 0 | Vvi-Vitvi16g00375\_t001 |  |  |  |  |  |  |  |  |  |  |  |  |
| 0 | Vvi-Vitvi16g01444\_t001 |  |  |  |  |  |  |  |  |  |  |  |  |
| 0 | Vvi-Vitvi16g01437\_t001 |  |  |  |  |  |  |  |  |  |  |  |  |
| 0 | Vvi-Vitvi16g01434\_t001 |  |  |  |  |  |  |  |  |  |  |  |  |
| 0 | Vvi-Vitvi16g01443\_t001 |  |  |  |  |  |  |  |  |  |  |  |  |
| 0 | Vvi-Vitvi16g01435\_t001 |  |  |  |  |  |  |  |  |  |  |  |  |
| 0 | Vvi-Vitvi16g04137\_t001 |  |  |  |  |  |  |  |  |  |  |  |  |
| 0 | Vvi-Vitvi16g00380\_t001 |  |  |  |  |  |  |  |  |  |  |  |  |
| 0 | Vvi-Vitvi16g04138\_t001 |  |  |  |  |  |  |  |  |  |  |  |  |
| 1 | Vvi-Vitvi16g00381\_t001 |  | Atr-ERN00215 |  |  |  |  |  |  |  |  |  |  |
| 1 | Vvi-Vitvi16g04139\_t001 |  | \| \| |  |  |  |  |  |  |  |  |  |  |
| 1 | Vvi-Vitvi16g00384\_t001 |  | \| \| |  |  |  |  |  |  |  |  |  |  |
| 1 | Vvi-Vitvi16g04140\_t001 |  | \| \| |  |  |  |  |  |  |  |  |  |  |
| 1 | Vvi-Vitvi16g00389\_t001 |  | \| \| |  |  |  |  |  |  |  |  |  |  |
| 1 | Vvi-Vitvi16g00390\_t001 |  | \| \| |  |  |  |  |  |  |  |  |  |  |
| 1 | Vvi-Vitvi16g04141\_t001 |  | \| \| |  |  |  |  |  |  |  |  |  |  |
| 1 | Vvi-Vitvi16g04142\_t001 |  | \| \| |  |  |  |  |  |  |  |  |  |  |
| 1 | Vvi-Vitvi16g00394\_t001 |  | \| \| |  |  |  |  |  |  |  |  |  |  |
| 1 | Vvi-Vitvi16g04143\_t001 |  | \| \| |  |  |  |  |  |  |  |  |  |  |
| 1 | Vvi-Vitvi16g04144\_t001 |  | \| \| |  |  |  |  |  |  |  |  |  |  |
| 1 | Vvi-Vitvi16g00404\_t001 |  | \| \| |  |  |  |  |  |  |  |  |  |  |
| 1 | Vvi-Vitvi16g00406\_t001 |  | \| \| |  |  |  |  |  |  |  |  |  |  |
| 1 | Vvi-Vitvi16g04145\_t001 |  | \| \| |  |  |  |  |  |  |  |  |  |  |
| 1 | Vvi-Vitvi16g04146\_t001 |  | \| \| |  |  |  |  |  |  |  |  |  |  |
| 1 | Vvi-Vitvi16g04147\_t001 |  | \| \| |  |  |  |  |  |  |  |  |  |  |
| 1 | Vvi-Vitvi16g04148\_t001 |  | \| \| |  |  |  |  |  |  |  |  |  |  |
| 1 | Vvi-Vitvi16g00413\_t001 |  | \| \| |  |  |  |  |  |  |  |  |  |  |
| 1 | Vvi-Vitvi16g00414\_t001 |  | \| \| |  |  |  |  |  |  |  |  |  |  |
| 1 | Vvi-Vitvi16g04149\_t001 |  | \| \| |  |  |  |  |  |  |  |  |  |  |
| 1 | Vvi-Vitvi16g04150\_t001 |  | \| \| |  |  |  |  |  |  |  |  |  |  |
| 1 | Vvi-Vitvi16g00418\_t001 |  | \| \| |  |  |  |  |  |  |  |  |  |  |
| 1 | Vvi-Vitvi16g01683\_t001 |  | \| \| |  |  |  |  |  |  |  |  |  |  |
| 1 | Vvi-Vitvi16g04151\_t001 |  | \| \| |  |  |  |  |  |  |  |  |  |  |
| 1 | Vvi-Vitvi16g04152\_t001 |  | \| \| |  |  |  |  |  |  |  |  |  |  |
| 1 | Vvi-Vitvi16g00427\_t001 |  | Atr-ERN00208 |  |  |  |  |  |  |  |  |  |  |
| 1 | Vvi-Vitvi16g01686\_t001 |  | \| \| |  |  |  |  |  |  |  |  |  |  |
| 1 | Vvi-Vitvi16g04153\_t001 |  | \| \| |  |  |  |  |  |  |  |  |  |  |
| 1 | Vvi-Vitvi16g00429\_t001 |  | Atr-ERN00207 |  |  |  |  |  |  |  |  |  |  |
| 1 | Vvi-Vitvi16g00433\_t001 |  | \| \| |  |  |  |  |  |  |  |  |  |  |
| 1 | Vvi-Vitvi16g01687\_t001 |  | \| \| |  |  |  |  |  |  |  |  |  |  |
| 1 | Vvi-Vitvi16g04154\_t001 |  | \| \| |  |  |  |  |  |  |  |  |  |  |
| 1 | Vvi-Vitvi16g00436\_t001 |  | Atr-ERN00203 |  |  |  |  |  |  |  |  |  |  |
| 1 | Vvi-Vitvi16g04155\_t001 |  | \| \| |  |  |  |  |  |  |  |  |  |  |
| 1 | Vvi-Vitvi16g04156\_t001 |  | \| \| |  |  |  |  |  |  |  |  |  |  |
| 1 | Vvi-Vitvi16g04157\_t001 |  | \| \| |  |  |  |  |  |  |  |  |  |  |
| 1 | Vvi-Vitvi16g00437\_t001 |  | Atr-ERN00195 |  |  |  |  |  |  |  |  |  |  |
| 1 | Vvi-Vitvi16g00439\_t003 |  | Atr-ERN00191 |  |  |  |  |  |  |  |  |  |  |
| 1 | Vvi-Vitvi16g00440\_t001 |  | \| \| |  |  |  |  |  |  |  |  |  |  |
| 1 | Vvi-Vitvi16g00441\_t001 |  | \| \| |  |  |  |  |  |  |  |  |  |  |
| 1 | Vvi-Vitvi16g00442\_t001 |  | \| \| |  |  |  |  |  |  |  |  |  |  |
| 1 | Vvi-Vitvi16g01689\_t001 |  | \| \| |  |  |  |  |  |  |  |  |  |  |
| 1 | Vvi-Vitvi16g00443\_t001 |  | \| \| |  |  |  |  |  |  |  |  |  |  |
| 1 | Vvi-Vitvi16g00444\_t001 |  | \| \| |  |  |  |  |  |  |  |  |  |  |
| 1 | Vvi-Vitvi16g04158\_t001 |  | \| \| |  |  |  |  |  |  |  |  |  |  |
| 1 | Vvi-Vitvi16g04159\_t001 |  | \| \| |  |  |  |  |  |  |  |  |  |  |
| 1 | Vvi-Vitvi16g04160\_t001 |  | \| \| |  |  |  |  |  |  |  |  |  |  |
| 1 | Vvi-Vitvi16g04161\_t001 |  | \| \| |  |  |  |  |  |  |  |  |  |  |
| 1 | Vvi-Vitvi16g04162\_t001 |  | \| \| |  |  |  |  |  |  |  |  |  |  |
| 1 | Vvi-Vitvi16g04163\_t001 |  | \| \| |  |  |  |  |  |  |  |  |  |  |
| 1 | Vvi-Vitvi16g04164\_t001 |  | \| \| |  |  |  |  |  |  |  |  |  |  |
| 2 | Vvi-Vitvi16g00455\_t001 |  | \| \| |  | Atr-ERN00176 |  |  |  |  |  |  |  |  |
| 2 | Vvi-Vitvi16g01695\_t001 |  | \| \| |  | \| \| |  |  |  |  |  |  |  |  |
| 2 | Vvi-Vitvi16g00458\_t001 |  | \| \| |  | \| \| |  |  |  |  |  |  |  |  |
| 2 | Vvi-Vitvi16g00460\_t001 |  | \| \| |  | \| \| |  |  |  |  |  |  |  |  |
| 2 | Vvi-Vitvi16g04165\_t001 |  | \| \| |  | \| \| |  |  |  |  |  |  |  |  |
| 2 | Vvi-Vitvi16g01697\_t001 |  | \| \| |  | \| \| |  |  |  |  |  |  |  |  |
| 2 | Vvi-Vitvi16g01698\_t001 |  | \| \| |  | \| \| |  |  |  |  |  |  |  |  |
| 2 | Vvi-Vitvi16g00461\_t001 |  | \| \| |  | \| \| |  |  |  |  |  |  |  |  |
| 2 | Vvi-Vitvi16g00462\_t001 |  | \| \| |  | \| \| |  |  |  |  |  |  |  |  |
| 2 | Vvi-Vitvi16g00463\_t001 |  | Atr-ERN00182 |  | Atr-ERN00182 |  |  |  |  |  |  |  |  |
| 2 | Vvi-Vitvi16g01699\_t001 |  | \| \| |  | \| \| |  |  |  |  |  |  |  |  |
| 2 | Vvi-Vitvi16g01701\_t001 |  | \| \| |  | \| \| |  |  |  |  |  |  |  |  |
| 2 | Vvi-Vitvi16g04166\_t001 |  | \| \| |  | \| \| |  |  |  |  |  |  |  |  |
| 2 | Vvi-Vitvi16g04167\_t001 |  | \| \| |  | \| \| |  |  |  |  |  |  |  |  |
| 2 | Vvi-Vitvi16g01703\_t001 |  | \| \| |  | \| \| |  |  |  |  |  |  |  |  |
| 2 | Vvi-Vitvi16g04168\_t001 |  | \| \| |  | \| \| |  |  |  |  |  |  |  |  |
| 2 | Vvi-Vitvi16g01707\_t001 |  | \| \| |  | \| \| |  |  |  |  |  |  |  |  |
| 2 | Vvi-Vitvi16g00474\_t001 |  | \| \| |  | Atr-ERN00183 |  |  |  |  |  |  |  |  |
| 2 | Vvi-Vitvi16g00475\_t001 |  | \| \| |  | Atr-ERN00189 |  |  |  |  |  |  |  |  |
| 2 | Vvi-Vitvi16g04169\_t001 |  | \| \| |  | \| \| |  |  |  |  |  |  |  |  |
| 2 | Vvi-Vitvi16g00477\_t001 |  | \| \| |  | Atr-ERN00190 |  |  |  |  |  |  |  |  |
| 2 | Vvi-Vitvi16g04170\_t001 |  | \| \| |  | \| \| |  |  |  |  |  |  |  |  |
| 2 | Vvi-Vitvi16g00479\_t001 |  | Atr-ERN00171 |  | \| \| |  |  |  |  |  |  |  |  |
| 2 | Vvi-Vitvi16g00481\_t001 |  | \| \| |  | \| \| |  |  |  |  |  |  |  |  |
| 2 | Vvi-Vitvi16g00486\_t001 |  | \| \| |  | \| \| |  |  |  |  |  |  |  |  |
| 2 | Vvi-Vitvi16g04171\_t001 |  | \| \| |  | \| \| |  |  |  |  |  |  |  |  |
| 2 | Vvi-Vitvi16g00497\_t001 |  | \| \| |  | \| \| |  |  |  |  |  |  |  |  |
| 2 | Vvi-Vitvi16g00499\_t001 |  | \| \| |  | \| \| |  |  |  |  |  |  |  |  |
| 2 | Vvi-Vitvi16g04172\_t001 |  | \| \| |  | \| \| |  |  |  |  |  |  |  |  |
| 2 | Vvi-Vitvi16g04173\_t001 |  | \| \| |  | \| \| |  |  |  |  |  |  |  |  |
| 2 | Vvi-Vitvi16g01710\_t001 |  | \| \| |  | \| \| |  |  |  |  |  |  |  |  |
| 2 | Vvi-Vitvi16g00501\_t001 |  | \| \| |  | \| \| |  |  |  |  |  |  |  |  |
| 2 | Vvi-Vitvi16g00502\_t001 |  | \| \| |  | \| \| |  |  |  |  |  |  |  |  |
| 2 | Vvi-Vitvi16g01711\_t001 |  | \| \| |  | Atr-ERN00192 |  |  |  |  |  |  |  |  |
| 1 | Vvi-Vitvi16g00504\_t001 |  | Atr-ERN00147 |  |  |  |  |  |  |  |  |  |  |
| 0 | Vvi-Vitvi16g04174\_t001 |  |  |  |  |  |  |  |  |  |  |  |  |
| 0 | Vvi-Vitvi16g04175\_t001 |  |  |  |  |  |  |  |  |  |  |  |  |
| 0 | Vvi-Vitvi16g00512\_t001 |  |  |  |  |  |  |  |  |  |  |  |  |
| 0 | Vvi-Vitvi16g04176\_t001 |  |  |  |  |  |  |  |  |  |  |  |  |
| 0 | Vvi-Vitvi16g04177\_t001 |  |  |  |  |  |  |  |  |  |  |  |  |
| 0 | Vvi-Vitvi16g04178\_t001 |  |  |  |  |  |  |  |  |  |  |  |  |
| 0 | Vvi-Vitvi16g04179\_t001 |  |  |  |  |  |  |  |  |  |  |  |  |
| 0 | Vvi-Vitvi16g00514\_t001 |  |  |  |  |  |  |  |  |  |  |  |  |
| 0 | Vvi-Vitvi16g04180\_t001 |  |  |  |  |  |  |  |  |  |  |  |  |
| 0 | Vvi-Vitvi16g01712\_t001 |  |  |  |  |  |  |  |  |  |  |  |  |
| 0 | Vvi-Vitvi16g00518\_t001 |  |  |  |  |  |  |  |  |  |  |  |  |
| 0 | Vvi-Vitvi16g00519\_t001 |  |  |  |  |  |  |  |  |  |  |  |  |
| 0 | Vvi-Vitvi16g00521\_t001 |  |  |  |  |  |  |  |  |  |  |  |  |
| 0 | Vvi-Vitvi16g00523\_t001 |  |  |  |  |  |  |  |  |  |  |  |  |
| 0 | Vvi-Vitvi16g00526\_t001 |  |  |  |  |  |  |  |  |  |  |  |  |
| 0 | Vvi-Vitvi16g00527\_t001 |  |  |  |  |  |  |  |  |  |  |  |  |
| 0 | Vvi-Vitvi16g04181\_t001 |  |  |  |  |  |  |  |  |  |  |  |  |
| 0 | Vvi-Vitvi16g04182\_t001 |  |  |  |  |  |  |  |  |  |  |  |  |
| 0 | Vvi-Vitvi16g04183\_t001 |  |  |  |  |  |  |  |  |  |  |  |  |
| 0 | Vvi-Vitvi16g04184\_t001 |  |  |  |  |  |  |  |  |  |  |  |  |
| 0 | Vvi-Vitvi16g04185\_t001 |  |  |  |  |  |  |  |  |  |  |  |  |
| 0 | Vvi-Vitvi16g04186\_t001 |  |  |  |  |  |  |  |  |  |  |  |  |
| 0 | Vvi-Vitvi16g04187\_t001 |  |  |  |  |  |  |  |  |  |  |  |  |
| 0 | Vvi-Vitvi16g04188\_t001 |  |  |  |  |  |  |  |  |  |  |  |  |
| 0 | Vvi-Vitvi16g04189\_t001 |  |  |  |  |  |  |  |  |  |  |  |  |
| 0 | Vvi-Vitvi16g04190\_t001 |  |  |  |  |  |  |  |  |  |  |  |  |
| 0 | Vvi-Vitvi16g04191\_t001 |  |  |  |  |  |  |  |  |  |  |  |  |
| 0 | Vvi-Vitvi16g04192\_t001 |  |  |  |  |  |  |  |  |  |  |  |  |
| 0 | Vvi-Vitvi16g02159\_t001 |  |  |  |  |  |  |  |  |  |  |  |  |
| 0 | Vvi-Vitvi16g04193\_t001 |  |  |  |  |  |  |  |  |  |  |  |  |
| 0 | Vvi-Vitvi16g04194\_t001 |  |  |  |  |  |  |  |  |  |  |  |  |
| 0 | Vvi-Vitvi16g02165\_t001 |  |  |  |  |  |  |  |  |  |  |  |  |
| 0 | Vvi-Vitvi16g04195\_t001 |  |  |  |  |  |  |  |  |  |  |  |  |
| 0 | Vvi-Vitvi16g04196\_t001 |  |  |  |  |  |  |  |  |  |  |  |  |
| 0 | Vvi-Vitvi16g04197\_t001 |  |  |  |  |  |  |  |  |  |  |  |  |
| 0 | Vvi-Vitvi16g02171\_t001 |  |  |  |  |  |  |  |  |  |  |  |  |
| 0 | Vvi-Vitvi16g02172\_t001 |  |  |  |  |  |  |  |  |  |  |  |  |
| 0 | Vvi-Vitvi16g04198\_t001 |  |  |  |  |  |  |  |  |  |  |  |  |
| 0 | Vvi-Vitvi16g04199\_t001 |  |  |  |  |  |  |  |  |  |  |  |  |
| 0 | Vvi-Vitvi16g04200\_t001 |  |  |  |  |  |  |  |  |  |  |  |  |
| 0 | Vvi-Vitvi16g04201\_t001 |  |  |  |  |  |  |  |  |  |  |  |  |
| 0 | Vvi-Vitvi16g04202\_t001 |  |  |  |  |  |  |  |  |  |  |  |  |
| 0 | Vvi-Vitvi16g04203\_t001 |  |  |  |  |  |  |  |  |  |  |  |  |
| 0 | Vvi-Vitvi16g04204\_t001 |  |  |  |  |  |  |  |  |  |  |  |  |
| 0 | Vvi-Vitvi16g04205\_t001 |  |  |  |  |  |  |  |  |  |  |  |  |
| 0 | Vvi-Vitvi16g04206\_t001 |  |  |  |  |  |  |  |  |  |  |  |  |
| 0 | Vvi-Vitvi16g04207\_t001 |  |  |  |  |  |  |  |  |  |  |  |  |
| 0 | Vvi-Vitvi16g04208\_t001 |  |  |  |  |  |  |  |  |  |  |  |  |
| 0 | Vvi-Vitvi16g00588\_t001 |  |  |  |  |  |  |  |  |  |  |  |  |
| 0 | Vvi-Vitvi16g04209\_t001 |  |  |  |  |  |  |  |  |  |  |  |  |
| 0 | Vvi-Vitvi16g00584\_t001 |  |  |  |  |  |  |  |  |  |  |  |  |
| 0 | Vvi-Vitvi16g04210\_t001 |  |  |  |  |  |  |  |  |  |  |  |  |
| 0 | Vvi-Vitvi16g04211\_t001 |  |  |  |  |  |  |  |  |  |  |  |  |
| 0 | Vvi-Vitvi16g00582\_t001 |  |  |  |  |  |  |  |  |  |  |  |  |
| 0 | Vvi-Vitvi16g04212\_t001 |  |  |  |  |  |  |  |  |  |  |  |  |
| 0 | Vvi-Vitvi16g00581\_t001 |  |  |  |  |  |  |  |  |  |  |  |  |
| 0 | Vvi-Vitvi16g01737\_t001 |  |  |  |  |  |  |  |  |  |  |  |  |
| 0 | Vvi-Vitvi16g04213\_t001 |  |  |  |  |  |  |  |  |  |  |  |  |
| 0 | Vvi-Vitvi16g00578\_t001 |  |  |  |  |  |  |  |  |  |  |  |  |
| 0 | Vvi-Vitvi16g00576\_t001 |  |  |  |  |  |  |  |  |  |  |  |  |
| 0 | Vvi-Vitvi16g04214\_t001 |  |  |  |  |  |  |  |  |  |  |  |  |
| 0 | Vvi-Vitvi16g04215\_t001 |  |  |  |  |  |  |  |  |  |  |  |  |
| 0 | Vvi-Vitvi16g00570\_t001 |  |  |  |  |  |  |  |  |  |  |  |  |
| 0 | Vvi-Vitvi16g04216\_t001 |  |  |  |  |  |  |  |  |  |  |  |  |
| 0 | Vvi-Vitvi16g00567\_t001 |  |  |  |  |  |  |  |  |  |  |  |  |
| 0 | Vvi-Vitvi16g04217\_t001 |  |  |  |  |  |  |  |  |  |  |  |  |
| 0 | Vvi-Vitvi16g04218\_t001 |  |  |  |  |  |  |  |  |  |  |  |  |
| 0 | Vvi-Vitvi16g01733\_t001 |  |  |  |  |  |  |  |  |  |  |  |  |
| 0 | Vvi-Vitvi16g00561\_t001 |  |  |  |  |  |  |  |  |  |  |  |  |
| 0 | Vvi-Vitvi16g04219\_t001 |  |  |  |  |  |  |  |  |  |  |  |  |
| 0 | Vvi-Vitvi16g01729\_t001 |  |  |  |  |  |  |  |  |  |  |  |  |
| 0 | Vvi-Vitvi16g04220\_t001 |  |  |  |  |  |  |  |  |  |  |  |  |
| 0 | Vvi-Vitvi16g04221\_t001 |  |  |  |  |  |  |  |  |  |  |  |  |
| 0 | Vvi-Vitvi16g01727\_t001 |  |  |  |  |  |  |  |  |  |  |  |  |
| 0 | Vvi-Vitvi16g01725\_t001 |  |  |  |  |  |  |  |  |  |  |  |  |
| 0 | Vvi-Vitvi16g00558\_t001 |  |  |  |  |  |  |  |  |  |  |  |  |
| 0 | Vvi-Vitvi16g04222\_t001 |  |  |  |  |  |  |  |  |  |  |  |  |
| 0 | Vvi-Vitvi16g01724\_t001 |  |  |  |  |  |  |  |  |  |  |  |  |
| 0 | Vvi-Vitvi16g01723\_t001 |  |  |  |  |  |  |  |  |  |  |  |  |
| 0 | Vvi-Vitvi16g04223\_t001 |  |  |  |  |  |  |  |  |  |  |  |  |
| 0 | Vvi-Vitvi16g00556\_t001 |  |  |  |  |  |  |  |  |  |  |  |  |
| 0 | Vvi-Vitvi16g00554\_t001 |  |  |  |  |  |  |  |  |  |  |  |  |
| 0 | Vvi-Vitvi16g04224\_t001 |  |  |  |  |  |  |  |  |  |  |  |  |
| 0 | Vvi-Vitvi16g04225\_t001 |  |  |  |  |  |  |  |  |  |  |  |  |
| 0 | Vvi-Vitvi16g01717\_t001 |  |  |  |  |  |  |  |  |  |  |  |  |
| 0 | Vvi-Vitvi16g00552\_t001 |  |  |  |  |  |  |  |  |  |  |  |  |
| 0 | Vvi-Vitvi16g04226\_t001 |  |  |  |  |  |  |  |  |  |  |  |  |
| 0 | Vvi-Vitvi16g04227\_t001 |  |  |  |  |  |  |  |  |  |  |  |  |
| 0 | Vvi-Vitvi16g00547\_t001 |  |  |  |  |  |  |  |  |  |  |  |  |
| 0 | Vvi-Vitvi16g04228\_t001 |  |  |  |  |  |  |  |  |  |  |  |  |
| 0 | Vvi-Vitvi16g00605\_t001 |  |  |  |  |  |  |  |  |  |  |  |  |
| 0 | Vvi-Vitvi16g00609\_t001 |  |  |  |  |  |  |  |  |  |  |  |  |
| 0 | Vvi-Vitvi16g04229\_t001 |  |  |  |  |  |  |  |  |  |  |  |  |
| 0 | Vvi-Vitvi16g04230\_t001 |  |  |  |  |  |  |  |  |  |  |  |  |
| 0 | Vvi-Vitvi16g04231\_t001 |  |  |  |  |  |  |  |  |  |  |  |  |
| 0 | Vvi-Vitvi16g04232\_t001 |  |  |  |  |  |  |  |  |  |  |  |  |
| 0 | Vvi-Vitvi16g04233\_t001 |  |  |  |  |  |  |  |  |  |  |  |  |
| 0 | Vvi-Vitvi16g04234\_t001 |  |  |  |  |  |  |  |  |  |  |  |  |
| 0 | Vvi-Vitvi16g04235\_t001 |  |  |  |  |  |  |  |  |  |  |  |  |
| 0 | Vvi-Vitvi16g04236\_t001 |  |  |  |  |  |  |  |  |  |  |  |  |
| 0 | Vvi-Vitvi16g04237\_t001 |  |  |  |  |  |  |  |  |  |  |  |  |
| 0 | Vvi-Vitvi16g04238\_t001 |  |  |  |  |  |  |  |  |  |  |  |  |
| 0 | Vvi-Vitvi16g04239\_t001 |  |  |  |  |  |  |  |  |  |  |  |  |
| 0 | Vvi-Vitvi16g04240\_t001 |  |  |  |  |  |  |  |  |  |  |  |  |
| 0 | Vvi-Vitvi16g01753\_t001 |  |  |  |  |  |  |  |  |  |  |  |  |
| 0 | Vvi-Vitvi16g04241\_t001 |  |  |  |  |  |  |  |  |  |  |  |  |
| 0 | Vvi-Vitvi16g04242\_t001 |  |  |  |  |  |  |  |  |  |  |  |  |
| 0 | Vvi-Vitvi16g04243\_t001 |  |  |  |  |  |  |  |  |  |  |  |  |
| 0 | Vvi-Vitvi16g04244\_t001 |  |  |  |  |  |  |  |  |  |  |  |  |
| 0 | Vvi-Vitvi16g04245\_t001 |  |  |  |  |  |  |  |  |  |  |  |  |
| 0 | Vvi-Vitvi16g04246\_t001 |  |  |  |  |  |  |  |  |  |  |  |  |
| 0 | Vvi-Vitvi16g04247\_t001 |  |  |  |  |  |  |  |  |  |  |  |  |
| 0 | Vvi-Vitvi16g04248\_t001 |  |  |  |  |  |  |  |  |  |  |  |  |
| 0 | Vvi-Vitvi16g04249\_t001 |  |  |  |  |  |  |  |  |  |  |  |  |
| 0 | Vvi-Vitvi16g04250\_t001 |  |  |  |  |  |  |  |  |  |  |  |  |
| 0 | Vvi-Vitvi16g04251\_t001 |  |  |  |  |  |  |  |  |  |  |  |  |
| 0 | Vvi-Vitvi16g04252\_t001 |  |  |  |  |  |  |  |  |  |  |  |  |
| 0 | Vvi-Vitvi16g04253\_t001 |  |  |  |  |  |  |  |  |  |  |  |  |
| 0 | Vvi-Vitvi16g04254\_t001 |  |  |  |  |  |  |  |  |  |  |  |  |
| 0 | Vvi-Vitvi16g00621\_t001 |  |  |  |  |  |  |  |  |  |  |  |  |
| 0 | Vvi-Vitvi16g04255\_t001 |  |  |  |  |  |  |  |  |  |  |  |  |
| 0 | Vvi-Vitvi16g04256\_t001 |  |  |  |  |  |  |  |  |  |  |  |  |
| 0 | Vvi-Vitvi16g00625\_t001 |  |  |  |  |  |  |  |  |  |  |  |  |
| 0 | Vvi-Vitvi16g04257\_t001 |  |  |  |  |  |  |  |  |  |  |  |  |
| 0 | Vvi-Vitvi16g04258\_t001 |  |  |  |  |  |  |  |  |  |  |  |  |
| 0 | Vvi-Vitvi16g04259\_t001 |  |  |  |  |  |  |  |  |  |  |  |  |
| 0 | Vvi-Vitvi16g00627\_t001 |  |  |  |  |  |  |  |  |  |  |  |  |
| 0 | Vvi-Vitvi16g04260\_t001 |  |  |  |  |  |  |  |  |  |  |  |  |
| 0 | Vvi-Vitvi16g00630\_t001 |  |  |  |  |  |  |  |  |  |  |  |  |
| 0 | Vvi-Vitvi16g04261\_t001 |  |  |  |  |  |  |  |  |  |  |  |  |
| 0 | Vvi-Vitvi16g04262\_t001 |  |  |  |  |  |  |  |  |  |  |  |  |
| 0 | Vvi-Vitvi16g04263\_t001 |  |  |  |  |  |  |  |  |  |  |  |  |
| 0 | Vvi-Vitvi16g01767\_t001 |  |  |  |  |  |  |  |  |  |  |  |  |
| 0 | Vvi-Vitvi16g04264\_t001 |  |  |  |  |  |  |  |  |  |  |  |  |
| 0 | Vvi-Vitvi16g04265\_t001 |  |  |  |  |  |  |  |  |  |  |  |  |
| 0 | Vvi-Vitvi16g04266\_t001 |  |  |  |  |  |  |  |  |  |  |  |  |
| 0 | Vvi-Vitvi16g01768\_t001 |  |  |  |  |  |  |  |  |  |  |  |  |
| 0 | Vvi-Vitvi16g01769\_t001 |  |  |  |  |  |  |  |  |  |  |  |  |
| 0 | Vvi-Vitvi16g01770\_t001 |  |  |  |  |  |  |  |  |  |  |  |  |
| 0 | Vvi-Vitvi16g00636\_t001 |  |  |  |  |  |  |  |  |  |  |  |  |
| 0 | Vvi-Vitvi16g04267\_t001 |  |  |  |  |  |  |  |  |  |  |  |  |
| 0 | Vvi-Vitvi16g04268\_t001 |  |  |  |  |  |  |  |  |  |  |  |  |
| 0 | Vvi-Vitvi16g04269\_t001 |  |  |  |  |  |  |  |  |  |  |  |  |
| 0 | Vvi-Vitvi16g00637\_t001 |  |  |  |  |  |  |  |  |  |  |  |  |
| 0 | Vvi-Vitvi16g04270\_t001 |  |  |  |  |  |  |  |  |  |  |  |  |
| 0 | Vvi-Vitvi16g04271\_t001 |  |  |  |  |  |  |  |  |  |  |  |  |
| 0 | Vvi-Vitvi16g04272\_t001 |  |  |  |  |  |  |  |  |  |  |  |  |
| 0 | Vvi-Vitvi16g01772\_t001 |  |  |  |  |  |  |  |  |  |  |  |  |
| 0 | Vvi-Vitvi16g04273\_t001 |  |  |  |  |  |  |  |  |  |  |  |  |
| 0 | Vvi-Vitvi16g04274\_t001 |  |  |  |  |  |  |  |  |  |  |  |  |
| 0 | Vvi-Vitvi16g04275\_t001 |  |  |  |  |  |  |  |  |  |  |  |  |
| 0 | Vvi-Vitvi16g00648\_t001 |  |  |  |  |  |  |  |  |  |  |  |  |
| 0 | Vvi-Vitvi16g01774\_t001 |  |  |  |  |  |  |  |  |  |  |  |  |
| 0 | Vvi-Vitvi16g01776\_t001 |  |  |  |  |  |  |  |  |  |  |  |  |
| 0 | Vvi-Vitvi16g00652\_t001 |  |  |  |  |  |  |  |  |  |  |  |  |
| 0 | Vvi-Vitvi16g00654\_t001 |  |  |  |  |  |  |  |  |  |  |  |  |
| 0 | Vvi-Vitvi16g00655\_t001 |  |  |  |  |  |  |  |  |  |  |  |  |
| 0 | Vvi-Vitvi16g00656\_t001 |  |  |  |  |  |  |  |  |  |  |  |  |
| 0 | Vvi-Vitvi16g00657\_t001 |  |  |  |  |  |  |  |  |  |  |  |  |
| 0 | Vvi-Vitvi16g00658\_t001 |  |  |  |  |  |  |  |  |  |  |  |  |
| 0 | Vvi-Vitvi16g00659\_t001 |  |  |  |  |  |  |  |  |  |  |  |  |
| 0 | Vvi-Vitvi16g00661\_t001 |  |  |  |  |  |  |  |  |  |  |  |  |
| 0 | Vvi-Vitvi16g00662\_t001 |  |  |  |  |  |  |  |  |  |  |  |  |
| 0 | Vvi-Vitvi16g00664\_t001 |  |  |  |  |  |  |  |  |  |  |  |  |
| 0 | Vvi-Vitvi16g04276\_t001 |  |  |  |  |  |  |  |  |  |  |  |  |
| 0 | Vvi-Vitvi16g00669\_t001 |  |  |  |  |  |  |  |  |  |  |  |  |
| 0 | Vvi-Vitvi16g00671\_t001 |  |  |  |  |  |  |  |  |  |  |  |  |
| 0 | Vvi-Vitvi16g00672\_t002 |  |  |  |  |  |  |  |  |  |  |  |  |
| 0 | Vvi-Vitvi16g00673\_t001 |  |  |  |  |  |  |  |  |  |  |  |  |
| 0 | Vvi-Vitvi16g04278\_t001 |  |  |  |  |  |  |  |  |  |  |  |  |
| 0 | Vvi-Vitvi16g00675\_t001 |  |  |  |  |  |  |  |  |  |  |  |  |
| 0 | Vvi-Vitvi16g04279\_t001 |  |  |  |  |  |  |  |  |  |  |  |  |
| 0 | Vvi-Vitvi16g00676\_t001 |  |  |  |  |  |  |  |  |  |  |  |  |
| 0 | Vvi-Vitvi16g04280\_t001 |  |  |  |  |  |  |  |  |  |  |  |  |
| 0 | Vvi-Vitvi16g04281\_t001 |  |  |  |  |  |  |  |  |  |  |  |  |
| 0 | Vvi-Vitvi16g04282\_t001 |  |  |  |  |  |  |  |  |  |  |  |  |
| 0 | Vvi-Vitvi16g00681\_t001 |  |  |  |  |  |  |  |  |  |  |  |  |
| 0 | Vvi-Vitvi16g04283\_t001 |  |  |  |  |  |  |  |  |  |  |  |  |
| 0 | Vvi-Vitvi16g00688\_t001 |  |  |  |  |  |  |  |  |  |  |  |  |
| 0 | Vvi-Vitvi16g00692\_t002 |  |  |  |  |  |  |  |  |  |  |  |  |
| 0 | Vvi-Vitvi16g00693\_t001 |  |  |  |  |  |  |  |  |  |  |  |  |
| 0 | Vvi-Vitvi16g01781\_t001 |  |  |  |  |  |  |  |  |  |  |  |  |
| 0 | Vvi-Vitvi16g04284\_t001 |  |  |  |  |  |  |  |  |  |  |  |  |
| 0 | Vvi-Vitvi16g01782\_t001 |  |  |  |  |  |  |  |  |  |  |  |  |
| 0 | Vvi-Vitvi16g04285\_t001 |  |  |  |  |  |  |  |  |  |  |  |  |
| 0 | Vvi-Vitvi16g04286\_t001 |  |  |  |  |  |  |  |  |  |  |  |  |
| 0 | Vvi-Vitvi16g04287\_t001 |  |  |  |  |  |  |  |  |  |  |  |  |
| 0 | Vvi-Vitvi16g01784\_t001 |  |  |  |  |  |  |  |  |  |  |  |  |
| 0 | Vvi-Vitvi16g04288\_t001 |  |  |  |  |  |  |  |  |  |  |  |  |
| 0 | Vvi-Vitvi16g01786\_t001 |  |  |  |  |  |  |  |  |  |  |  |  |
| 0 | Vvi-Vitvi16g04289\_t001 |  |  |  |  |  |  |  |  |  |  |  |  |
| 0 | Vvi-Vitvi16g00703\_t001 |  |  |  |  |  |  |  |  |  |  |  |  |
| 0 | Vvi-Vitvi16g00706\_t001 |  |  |  |  |  |  |  |  |  |  |  |  |
| 0 | Vvi-Vitvi16g04290\_t001 |  |  |  |  |  |  |  |  |  |  |  |  |
| 0 | Vvi-Vitvi16g00707\_t001 |  |  |  |  |  |  |  |  |  |  |  |  |
| 0 | Vvi-Vitvi16g00709\_t001 |  |  |  |  |  |  |  |  |  |  |  |  |
| 0 | Vvi-Vitvi16g00710\_t001 |  |  |  |  |  |  |  |  |  |  |  |  |
| 0 | Vvi-Vitvi16g00711\_t001 |  |  |  |  |  |  |  |  |  |  |  |  |
| 0 | Vvi-Vitvi16g04291\_t001 |  |  |  |  |  |  |  |  |  |  |  |  |
| 0 | Vvi-Vitvi16g00712\_t001 |  |  |  |  |  |  |  |  |  |  |  |  |
| 0 | Vvi-Vitvi16g00713\_t001 |  |  |  |  |  |  |  |  |  |  |  |  |
| 0 | Vvi-Vitvi16g00715\_t001 |  |  |  |  |  |  |  |  |  |  |  |  |
| 0 | Vvi-Vitvi16g00716\_t001 |  |  |  |  |  |  |  |  |  |  |  |  |
| 0 | Vvi-Vitvi16g00717\_t001 |  |  |  |  |  |  |  |  |  |  |  |  |
| 0 | Vvi-Vitvi16g00719\_t001 |  |  |  |  |  |  |  |  |  |  |  |  |
| 0 | Vvi-Vitvi16g00720\_t001 |  |  |  |  |  |  |  |  |  |  |  |  |
| 0 | Vvi-Vitvi16g01789\_t001 |  |  |  |  |  |  |  |  |  |  |  |  |
| 0 | Vvi-Vitvi16g00723\_t001 |  |  |  |  |  |  |  |  |  |  |  |  |
| 0 | Vvi-Vitvi16g00724\_t001 |  |  |  |  |  |  |  |  |  |  |  |  |
| 0 | Vvi-Vitvi16g01790\_t001 |  |  |  |  |  |  |  |  |  |  |  |  |
| 0 | Vvi-Vitvi16g01791\_t001 |  |  |  |  |  |  |  |  |  |  |  |  |
| 0 | Vvi-Vitvi16g00725\_t001 |  |  |  |  |  |  |  |  |  |  |  |  |
| 0 | Vvi-Vitvi16g04292\_t001 |  |  |  |  |  |  |  |  |  |  |  |  |
| 0 | Vvi-Vitvi16g04293\_t001 |  |  |  |  |  |  |  |  |  |  |  |  |
| 0 | Vvi-Vitvi16g00729\_t001 |  |  |  |  |  |  |  |  |  |  |  |  |
| 0 | Vvi-Vitvi16g04294\_t001 |  |  |  |  |  |  |  |  |  |  |  |  |
| 0 | Vvi-Vitvi16g04295\_t001 |  |  |  |  |  |  |  |  |  |  |  |  |
| 0 | Vvi-Vitvi16g04296\_t001 |  |  |  |  |  |  |  |  |  |  |  |  |
| 0 | Vvi-Vitvi16g00733\_t001 |  |  |  |  |  |  |  |  |  |  |  |  |
| 0 | Vvi-Vitvi16g01795\_t001 |  |  |  |  |  |  |  |  |  |  |  |  |
| 0 | Vvi-Vitvi16g00735\_t001 |  |  |  |  |  |  |  |  |  |  |  |  |
| 0 | Vvi-Vitvi16g04297\_t001 |  |  |  |  |  |  |  |  |  |  |  |  |
| 0 | Vvi-Vitvi16g00741\_t001 |  |  |  |  |  |  |  |  |  |  |  |  |
| 0 | Vvi-Vitvi16g01796\_t001 |  |  |  |  |  |  |  |  |  |  |  |  |
| 0 | Vvi-Vitvi16g00744\_t001 |  |  |  |  |  |  |  |  |  |  |  |  |
| 0 | Vvi-Vitvi16g01800\_t001 |  |  |  |  |  |  |  |  |  |  |  |  |
| 0 | Vvi-Vitvi16g00745\_t001 |  |  |  |  |  |  |  |  |  |  |  |  |
| 0 | Vvi-Vitvi16g00746\_t003 |  |  |  |  |  |  |  |  |  |  |  |  |
| 0 | Vvi-Vitvi16g00747\_t001 |  |  |  |  |  |  |  |  |  |  |  |  |
| 0 | Vvi-Vitvi16g04298\_t001 |  |  |  |  |  |  |  |  |  |  |  |  |
| 0 | Vvi-Vitvi16g00750\_t001 |  |  |  |  |  |  |  |  |  |  |  |  |
| 0 | Vvi-Vitvi16g00752\_t001 |  |  |  |  |  |  |  |  |  |  |  |  |
| 0 | Vvi-Vitvi16g00753\_t001 |  |  |  |  |  |  |  |  |  |  |  |  |
| 0 | Vvi-Vitvi16g01450\_t001 |  |  |  |  |  |  |  |  |  |  |  |  |
| 0 | Vvi-Vitvi16g04299\_t001 |  |  |  |  |  |  |  |  |  |  |  |  |
| 0 | Vvi-Vitvi16g00759\_t001 |  |  |  |  |  |  |  |  |  |  |  |  |
| 0 | Vvi-Vitvi16g04300\_t001 |  |  |  |  |  |  |  |  |  |  |  |  |
| 0 | Vvi-Vitvi16g04301\_t001 |  |  |  |  |  |  |  |  |  |  |  |  |
| 0 | Vvi-Vitvi16g00765\_t001 |  |  |  |  |  |  |  |  |  |  |  |  |
| 0 | Vvi-Vitvi16g00766\_t001 |  |  |  |  |  |  |  |  |  |  |  |  |
| 0 | Vvi-Vitvi16g04302\_t001 |  |  |  |  |  |  |  |  |  |  |  |  |
| 0 | Vvi-Vitvi16g04303\_t001 |  |  |  |  |  |  |  |  |  |  |  |  |
| 0 | Vvi-Vitvi16g00773\_t001 |  |  |  |  |  |  |  |  |  |  |  |  |
| 0 | Vvi-Vitvi16g04304\_t001 |  |  |  |  |  |  |  |  |  |  |  |  |
| 0 | Vvi-Vitvi16g00775\_t001 |  |  |  |  |  |  |  |  |  |  |  |  |
| 0 | Vvi-Vitvi16g00777\_t001 |  |  |  |  |  |  |  |  |  |  |  |  |
| 0 | Vvi-Vitvi16g00779\_t001 |  |  |  |  |  |  |  |  |  |  |  |  |
| 0 | Vvi-Vitvi16g04305\_t001 |  |  |  |  |  |  |  |  |  |  |  |  |
| 0 | Vvi-Vitvi16g00782\_t001 |  |  |  |  |  |  |  |  |  |  |  |  |
| 0 | Vvi-Vitvi16g00783\_t001 |  |  |  |  |  |  |  |  |  |  |  |  |
| 0 | Vvi-Vitvi16g04306\_t001 |  |  |  |  |  |  |  |  |  |  |  |  |
| 0 | Vvi-Vitvi16g04307\_t001 |  |  |  |  |  |  |  |  |  |  |  |  |
| 0 | Vvi-Vitvi16g04308\_t001 |  |  |  |  |  |  |  |  |  |  |  |  |
| 0 | Vvi-Vitvi16g04309\_t001 |  |  |  |  |  |  |  |  |  |  |  |  |
| 0 | Vvi-Vitvi16g01808\_t001 |  |  |  |  |  |  |  |  |  |  |  |  |
| 0 | Vvi-Vitvi16g04310\_t001 |  |  |  |  |  |  |  |  |  |  |  |  |
| 0 | Vvi-Vitvi16g04311\_t001 |  |  |  |  |  |  |  |  |  |  |  |  |
| 0 | Vvi-Vitvi16g01809\_t001 |  |  |  |  |  |  |  |  |  |  |  |  |
| 0 | Vvi-Vitvi16g00787\_t001 |  |  |  |  |  |  |  |  |  |  |  |  |
| 0 | Vvi-Vitvi16g00788\_t001 |  |  |  |  |  |  |  |  |  |  |  |  |
| 0 | Vvi-Vitvi16g04312\_t001 |  |  |  |  |  |  |  |  |  |  |  |  |
| 0 | Vvi-Vitvi16g00789\_t001 |  |  |  |  |  |  |  |  |  |  |  |  |
| 0 | Vvi-Vitvi16g00790\_t001 |  |  |  |  |  |  |  |  |  |  |  |  |
| 0 | Vvi-Vitvi16g00791\_t001 |  |  |  |  |  |  |  |  |  |  |  |  |
| 0 | Vvi-Vitvi16g00792\_t001 |  |  |  |  |  |  |  |  |  |  |  |  |
| 0 | Vvi-Vitvi16g00793\_t001 |  |  |  |  |  |  |  |  |  |  |  |  |
| 0 | Vvi-Vitvi16g00794\_t001 |  |  |  |  |  |  |  |  |  |  |  |  |
| 0 | Vvi-Vitvi16g00796\_t001 |  |  |  |  |  |  |  |  |  |  |  |  |
| 0 | Vvi-Vitvi16g00797\_t001 |  |  |  |  |  |  |  |  |  |  |  |  |
| 0 | Vvi-Vitvi16g00798\_t001 |  |  |  |  |  |  |  |  |  |  |  |  |
| 0 | Vvi-Vitvi16g01818\_t001 |  |  |  |  |  |  |  |  |  |  |  |  |
| 0 | Vvi-Vitvi16g04313\_t001 |  |  |  |  |  |  |  |  |  |  |  |  |
| 0 | Vvi-Vitvi16g04314\_t001 |  |  |  |  |  |  |  |  |  |  |  |  |
| 0 | Vvi-Vitvi16g04315\_t001 |  |  |  |  |  |  |  |  |  |  |  |  |
| 0 | Vvi-Vitvi16g04316\_t001 |  |  |  |  |  |  |  |  |  |  |  |  |
| 0 | Vvi-Vitvi16g04317\_t001 |  |  |  |  |  |  |  |  |  |  |  |  |
| 0 | Vvi-Vitvi16g00803\_t001 |  |  |  |  |  |  |  |  |  |  |  |  |
| 0 | Vvi-Vitvi16g00804\_t001 |  |  |  |  |  |  |  |  |  |  |  |  |
| 0 | Vvi-Vitvi16g00805\_t001 |  |  |  |  |  |  |  |  |  |  |  |  |
| 0 | Vvi-Vitvi16g00806\_t001 |  |  |  |  |  |  |  |  |  |  |  |  |
| 0 | Vvi-Vitvi16g00807\_t001 |  |  |  |  |  |  |  |  |  |  |  |  |
| 0 | Vvi-Vitvi16g00808\_t001 |  |  |  |  |  |  |  |  |  |  |  |  |
| 1 | Vvi-Vitvi16g00809\_t001 |  | Atr-ERN13335 |  |  |  |  |  |  |  |  |  |  |
| 1 | Vvi-Vitvi16g00811\_t001 |  | \| \| |  |  |  |  |  |  |  |  |  |  |
| 1 | Vvi-Vitvi16g04318\_t001 |  | \| \| |  |  |  |  |  |  |  |  |  |  |
| 1 | Vvi-Vitvi16g04319\_t001 |  | \| \| |  |  |  |  |  |  |  |  |  |  |
| 1 | Vvi-Vitvi16g00816\_t001 |  | \| \| |  |  |  |  |  |  |  |  |  |  |
| 1 | Vvi-Vitvi16g00817\_t002 |  | Atr-ERN13315 |  |  |  |  |  |  |  |  |  |  |
| 1 | Vvi-Vitvi16g04320\_t001 |  | \| \| |  |  |  |  |  |  |  |  |  |  |
| 1 | Vvi-Vitvi16g00825\_t001 |  | \| \| |  |  |  |  |  |  |  |  |  |  |
| 1 | Vvi-Vitvi16g00826\_t001 |  | Atr-ERN13311 |  |  |  |  |  |  |  |  |  |  |
| 1 | Vvi-Vitvi16g00830\_t001 |  | Atr-ERN13302 |  |  |  |  |  |  |  |  |  |  |
| 1 | Vvi-Vitvi16g00831\_t001 |  | Atr-ERN13298 |  |  |  |  |  |  |  |  |  |  |
| 1 | Vvi-Vitvi16g00832\_t001 |  | \| \| |  |  |  |  |  |  |  |  |  |  |
| 1 | Vvi-Vitvi16g00833\_t001 |  | \| \| |  |  |  |  |  |  |  |  |  |  |
| 1 | Vvi-Vitvi16g04321\_t001 |  | \| \| |  |  |  |  |  |  |  |  |  |  |
| 1 | Vvi-Vitvi16g00834\_t001 |  | \| \| |  |  |  |  |  |  |  |  |  |  |
| 1 | Vvi-Vitvi16g01823\_t001 |  | \| \| |  |  |  |  |  |  |  |  |  |  |
| 1 | Vvi-Vitvi16g00835\_t001 |  | Atr-ERN13289 |  |  |  |  |  |  |  |  |  |  |
| 1 | Vvi-Vitvi16g00836\_t001 |  | Atr-ERN13288 |  |  |  |  |  |  |  |  |  |  |
| 1 | Vvi-Vitvi16g00837\_t002 |  | \| \| |  |  |  |  |  |  |  |  |  |  |
| 1 | Vvi-Vitvi16g00838\_t001 |  | \| \| |  |  |  |  |  |  |  |  |  |  |
| 1 | Vvi-Vitvi16g04322\_t001 |  | \| \| |  |  |  |  |  |  |  |  |  |  |
| 1 | Vvi-Vitvi16g00841\_t001 |  | Atr-ERN13281 |  |  |  |  |  |  |  |  |  |  |
| 1 | Vvi-Vitvi16g00843\_t001 |  | Atr-ERN13280 |  |  |  |  |  |  |  |  |  |  |
| 0 | Vvi-Vitvi16g04323\_t001 |  |  |  |  |  |  |  |  |  |  |  |  |
| 0 | Vvi-Vitvi16g00844\_t001 |  |  |  |  |  |  |  |  |  |  |  |  |
| 0 | Vvi-Vitvi16g00845\_t001 |  |  |  |  |  |  |  |  |  |  |  |  |
| 0 | Vvi-Vitvi16g00846\_t001 |  |  |  |  |  |  |  |  |  |  |  |  |
| 0 | Vvi-Vitvi16g00847\_t001 |  |  |  |  |  |  |  |  |  |  |  |  |
| 0 | Vvi-Vitvi16g00848\_t001 |  |  |  |  |  |  |  |  |  |  |  |  |
| 0 | Vvi-Vitvi16g00849\_t001 |  |  |  |  |  |  |  |  |  |  |  |  |
| 0 | Vvi-Vitvi16g00851\_t001 |  |  |  |  |  |  |  |  |  |  |  |  |
| 0 | Vvi-Vitvi16g00852\_t001 |  |  |  |  |  |  |  |  |  |  |  |  |
| 1 | Vvi-Vitvi16g00853\_t001 |  | Atr-ERN19494 |  |  |  |  |  |  |  |  |  |  |
| 1 | Vvi-Vitvi16g00854\_t001 |  | \| \| |  |  |  |  |  |  |  |  |  |  |
| 1 | Vvi-Vitvi16g04324\_t001 |  | \| \| |  |  |  |  |  |  |  |  |  |  |
| 1 | Vvi-Vitvi16g00855\_t001 |  | Atr-ERN19493 |  |  |  |  |  |  |  |  |  |  |
| 1 | Vvi-Vitvi16g00856\_t001 |  | Atr-ERN19491 |  |  |  |  |  |  |  |  |  |  |
| 1 | Vvi-Vitvi16g00857\_t001 |  | \| \| |  |  |  |  |  |  |  |  |  |  |
| 1 | Vvi-Vitvi16g00858\_t001 |  | \| \| |  |  |  |  |  |  |  |  |  |  |
| 1 | Vvi-Vitvi16g00859\_t001 |  | \| \| |  |  |  |  |  |  |  |  |  |  |
| 1 | Vvi-Vitvi16g04325\_t001 |  | \| \| |  |  |  |  |  |  |  |  |  |  |
| 1 | Vvi-Vitvi16g04326\_t001 |  | \| \| |  |  |  |  |  |  |  |  |  |  |
| 1 | Vvi-Vitvi16g00860\_t003 |  | Atr-ERN19484 |  |  |  |  |  |  |  |  |  |  |
| 1 | Vvi-Vitvi16g00863\_t001 |  | Atr-ERN19481 |  |  |  |  |  |  |  |  |  |  |
| 1 | Vvi-Vitvi16g00864\_t001 |  | Atr-ERN19480 |  |  |  |  |  |  |  |  |  |  |
| 1 | Vvi-Vitvi16g00866\_t001 |  | \| \| |  |  |  |  |  |  |  |  |  |  |
| 1 | Vvi-Vitvi16g00867\_t001 |  | \| \| |  |  |  |  |  |  |  |  |  |  |
| 1 | Vvi-Vitvi16g00869\_t001 |  | Atr-ERN19473 |  |  |  |  |  |  |  |  |  |  |
| 1 | Vvi-Vitvi16g00870\_t001 |  | Atr-ERN19466 |  |  |  |  |  |  |  |  |  |  |
| 1 | Vvi-Vitvi16g00871\_t001 |  | \| \| |  |  |  |  |  |  |  |  |  |  |
| 1 | Vvi-Vitvi16g00872\_t001 |  | \| \| |  |  |  |  |  |  |  |  |  |  |
| 1 | Vvi-Vitvi16g00874\_t001 |  | \| \| |  |  |  |  |  |  |  |  |  |  |
| 1 | Vvi-Vitvi16g04327\_t001 |  | \| \| |  |  |  |  |  |  |  |  |  |  |
| 1 | Vvi-Vitvi16g00875\_t001 |  | Atr-ERN19457 |  |  |  |  |  |  |  |  |  |  |
| 1 | Vvi-Vitvi16g00876\_t001 |  | \| \| |  |  |  |  |  |  |  |  |  |  |
| 1 | Vvi-Vitvi16g04328\_t001 |  | \| \| |  |  |  |  |  |  |  |  |  |  |
| 1 | Vvi-Vitvi16g00877\_t001 |  | Atr-ERN19455 |  |  |  |  |  |  |  |  |  |  |
| 1 | Vvi-Vitvi16g00878\_t001 |  | Atr-ERN19452 |  |  |  |  |  |  |  |  |  |  |
| 1 | Vvi-Vitvi16g00879\_t001 |  | \| \| |  |  |  |  |  |  |  |  |  |  |
| 1 | Vvi-Vitvi16g04329\_t001 |  | \| \| |  |  |  |  |  |  |  |  |  |  |
| 1 | Vvi-Vitvi16g01826\_t001 |  | \| \| |  |  |  |  |  |  |  |  |  |  |
| 1 | Vvi-Vitvi16g00880\_t001 |  | \| \| |  |  |  |  |  |  |  |  |  |  |
| 1 | Vvi-Vitvi16g00882\_t001 |  | \| \| |  |  |  |  |  |  |  |  |  |  |
| 1 | Vvi-Vitvi16g00883\_t001 |  | \| \| |  |  |  |  |  |  |  |  |  |  |
| 1 | Vvi-Vitvi16g00885\_t001 |  | \| \| |  |  |  |  |  |  |  |  |  |  |
| 1 | Vvi-Vitvi16g00886\_t001 |  | \| \| |  |  |  |  |  |  |  |  |  |  |
| 1 | Vvi-Vitvi16g00887\_t001 |  | \| \| |  |  |  |  |  |  |  |  |  |  |
| 1 | Vvi-Vitvi16g00889\_t001 |  | \| \| |  |  |  |  |  |  |  |  |  |  |
| 1 | Vvi-Vitvi16g00890\_t001 |  | \| \| |  |  |  |  |  |  |  |  |  |  |
| 1 | Vvi-Vitvi16g00891\_t001 |  | \| \| |  |  |  |  |  |  |  |  |  |  |
| 1 | Vvi-Vitvi16g00894\_t001 |  | \| \| |  |  |  |  |  |  |  |  |  |  |
| 1 | Vvi-Vitvi16g00895\_t001 |  | \| \| |  |  |  |  |  |  |  |  |  |  |
| 1 | Vvi-Vitvi16g00896\_t002 |  | \| \| |  |  |  |  |  |  |  |  |  |  |
| 1 | Vvi-Vitvi16g00897\_t001 |  | \| \| |  |  |  |  |  |  |  |  |  |  |
| 1 | Vvi-Vitvi16g00898\_t001 |  | \| \| |  |  |  |  |  |  |  |  |  |  |
| 1 | Vvi-Vitvi16g01827\_t001 |  | \| \| |  |  |  |  |  |  |  |  |  |  |
| 1 | Vvi-Vitvi16g00899\_t001 |  | Atr-ERN19433 |  |  |  |  |  |  |  |  |  |  |
| 1 | Vvi-Vitvi16g04330\_t001 |  | \| \| |  |  |  |  |  |  |  |  |  |  |
| 1 | Vvi-Vitvi16g00900\_t001 |  | \| \| |  |  |  |  |  |  |  |  |  |  |
| 1 | Vvi-Vitvi16g04331\_t001 |  | \| \| |  |  |  |  |  |  |  |  |  |  |
| 1 | Vvi-Vitvi16g00901\_t001 |  | \| \| |  |  |  |  |  |  |  |  |  |  |
| 1 | Vvi-Vitvi16g04332\_t001 |  | \| \| |  |  |  |  |  |  |  |  |  |  |
| 1 | Vvi-Vitvi16g01838\_t001 |  | \| \| |  |  |  |  |  |  |  |  |  |  |
| 1 | Vvi-Vitvi16g01839\_t001 |  | \| \| |  |  |  |  |  |  |  |  |  |  |
| 1 | Vvi-Vitvi16g04333\_t001 |  | \| \| |  |  |  |  |  |  |  |  |  |  |
| 1 | Vvi-Vitvi16g01842\_t001 |  | \| \| |  |  |  |  |  |  |  |  |  |  |
| 1 | Vvi-Vitvi16g01843\_t001 |  | \| \| |  |  |  |  |  |  |  |  |  |  |
| 1 | Vvi-Vitvi16g00912\_t001 |  | \| \| |  |  |  |  |  |  |  |  |  |  |
| 1 | Vvi-Vitvi16g00913\_t001 |  | \| \| |  |  |  |  |  |  |  |  |  |  |
| 1 | Vvi-Vitvi16g01844\_t001 |  | Atr-ERN19429 |  |  |  |  |  |  |  |  |  |  |
| 1 | Vvi-Vitvi16g01846\_t001 |  | Atr-ERN19424 |  |  |  |  |  |  |  |  |  |  |
| 1 | Vvi-Vitvi16g00915\_t001 |  | \| \| |  |  |  |  |  |  |  |  |  |  |
| 1 | Vvi-Vitvi16g01847\_t001 |  | \| \| |  |  |  |  |  |  |  |  |  |  |
| 1 | Vvi-Vitvi16g04334\_t001 |  | \| \| |  |  |  |  |  |  |  |  |  |  |
| 1 | Vvi-Vitvi16g04335\_t001 |  | \| \| |  |  |  |  |  |  |  |  |  |  |
| 1 | Vvi-Vitvi16g04336\_t001 |  | \| \| |  |  |  |  |  |  |  |  |  |  |
| 1 | Vvi-Vitvi16g00918\_t001 |  | \| \| |  |  |  |  |  |  |  |  |  |  |
| 1 | Vvi-Vitvi16g01849\_t001 |  | \| \| |  |  |  |  |  |  |  |  |  |  |
| 1 | Vvi-Vitvi16g04337\_t001 |  | \| \| |  |  |  |  |  |  |  |  |  |  |
| 1 | Vvi-Vitvi16g00919\_t001 |  | Atr-ERN19421 |  |  |  |  |  |  |  |  |  |  |
| 1 | Vvi-Vitvi16g01850\_t001 |  | \| \| |  |  |  |  |  |  |  |  |  |  |
| 1 | Vvi-Vitvi16g02150\_t001 |  | \| \| |  |  |  |  |  |  |  |  |  |  |
| 1 | Vvi-Vitvi16g01852\_t001 |  | \| \| |  |  |  |  |  |  |  |  |  |  |
| 1 | Vvi-Vitvi16g04338\_t001 |  | \| \| |  |  |  |  |  |  |  |  |  |  |
| 1 | Vvi-Vitvi16g00920\_t001 |  | \| \| |  |  |  |  |  |  |  |  |  |  |
| 1 | Vvi-Vitvi16g04339\_t001 |  | \| \| |  |  |  |  |  |  |  |  |  |  |
| 1 | Vvi-Vitvi16g00921\_t001 |  | \| \| |  |  |  |  |  |  |  |  |  |  |
| 1 | Vvi-Vitvi16g00922\_t001 |  | Atr-ERN19418 |  |  |  |  |  |  |  |  |  |  |
| 1 | Vvi-Vitvi16g01854\_t001 |  | Atr-ERN19412 |  |  |  |  |  |  |  |  |  |  |
| 1 | Vvi-Vitvi16g00926\_t001 |  | Atr-ERN19405 |  |  |  |  |  |  |  |  |  |  |
| 1 | Vvi-Vitvi16g00928\_t001 |  | \| \| |  |  |  |  |  |  |  |  |  |  |
| 1 | Vvi-Vitvi16g01857\_t006 |  | Atr-ERN19400 |  |  |  |  |  |  |  |  |  |  |
| 1 | Vvi-Vitvi16g00929\_t001 |  | \| \| |  |  |  |  |  |  |  |  |  |  |
| 1 | Vvi-Vitvi16g00931\_t001 |  | \| \| |  |  |  |  |  |  |  |  |  |  |
| 1 | Vvi-Vitvi16g01859\_t001 |  | \| \| |  |  |  |  |  |  |  |  |  |  |
| 1 | Vvi-Vitvi16g00933\_t001 |  | \| \| |  |  |  |  |  |  |  |  |  |  |
| 1 | Vvi-Vitvi16g00934\_t001 |  | Atr-ERN19393 |  |  |  |  |  |  |  |  |  |  |
| 1 | Vvi-Vitvi16g00935\_t001 |  | \| \| |  |  |  |  |  |  |  |  |  |  |
| 1 | Vvi-Vitvi16g00936\_t003 |  | Atr-ERN19391 |  |  |  |  |  |  |  |  |  |  |
| 1 | Vvi-Vitvi16g00937\_t001 |  | Atr-ERN19389 |  |  |  |  |  |  |  |  |  |  |
| 1 | Vvi-Vitvi16g00938\_t001 |  | \| \| |  |  |  |  |  |  |  |  |  |  |
| 1 | Vvi-Vitvi16g04340\_t001 |  | Atr-ERN19383 |  |  |  |  |  |  |  |  |  |  |
| 1 | Vvi-Vitvi16g01860\_t001 |  | \| \| |  |  |  |  |  |  |  |  |  |  |
| 1 | Vvi-Vitvi16g00942\_t001 |  | Atr-ERN19382 |  |  |  |  |  |  |  |  |  |  |
| 1 | Vvi-Vitvi16g00944\_t001 |  | Atr-ERN19379 |  |  |  |  |  |  |  |  |  |  |
| 1 | Vvi-Vitvi16g00946\_t001 |  | \| \| |  |  |  |  |  |  |  |  |  |  |
| 1 | Vvi-Vitvi16g00947\_t001 |  | \| \| |  |  |  |  |  |  |  |  |  |  |
| 1 | Vvi-Vitvi16g00948\_t001 |  | \| \| |  |  |  |  |  |  |  |  |  |  |
| 1 | Vvi-Vitvi16g01446\_t001 |  | \| \| |  |  |  |  |  |  |  |  |  |  |
| 1 | Vvi-Vitvi16g00949\_t004 |  | Atr-ERN19371 |  |  |  |  |  |  |  |  |  |  |
| 1 | Vvi-Vitvi16g00951\_t001 |  | Atr-ERN19367 |  |  |  |  |  |  |  |  |  |  |
| 1 | Vvi-Vitvi16g00952\_t001 |  | Atr-ERN19364 |  |  |  |  |  |  |  |  |  |  |
| 1 | Vvi-Vitvi16g00953\_t001 |  | Atr-ERN19362 |  |  |  |  |  |  |  |  |  |  |
| 1 | Vvi-Vitvi16g04341\_t001 |  | \| \| |  |  |  |  |  |  |  |  |  |  |
| 1 | Vvi-Vitvi16g00955\_t001 |  | Atr-ERN19361 |  |  |  |  |  |  |  |  |  |  |
| 1 | Vvi-Vitvi16g04342\_t001 |  | \| \| |  |  |  |  |  |  |  |  |  |  |
| 1 | Vvi-Vitvi16g00956\_t001 |  | Atr-ERN19357 |  |  |  |  |  |  |  |  |  |  |
| 1 | Vvi-Vitvi16g01861\_t001 |  | Atr-ERN19356 |  |  |  |  |  |  |  |  |  |  |
| 1 | Vvi-Vitvi16g00957\_t001 |  | Atr-ERN19355 |  |  |  |  |  |  |  |  |  |  |
| 1 | Vvi-Vitvi16g00959\_t002 |  | Atr-ERN19353 |  |  |  |  |  |  |  |  |  |  |
| 1 | Vvi-Vitvi16g00960\_t001 |  | \| \| |  |  |  |  |  |  |  |  |  |  |
| 1 | Vvi-Vitvi16g00961\_t001 |  | \| \| |  |  |  |  |  |  |  |  |  |  |
| 1 | Vvi-Vitvi16g01863\_t001 |  | \| \| |  |  |  |  |  |  |  |  |  |  |
| 1 | Vvi-Vitvi16g00962\_t001 |  | \| \| |  |  |  |  |  |  |  |  |  |  |
| 1 | Vvi-Vitvi16g00964\_t001 |  | Atr-ERN19350 |  |  |  |  |  |  |  |  |  |  |
| 1 | Vvi-Vitvi16g01864\_t001 |  | Atr-ERN19349 |  |  |  |  |  |  |  |  |  |  |
| 1 | Vvi-Vitvi16g04343\_t001 |  | \| \| |  |  |  |  |  |  |  |  |  |  |
| 1 | Vvi-Vitvi16g01866\_t001 |  | \| \| |  |  |  |  |  |  |  |  |  |  |
| 1 | Vvi-Vitvi16g00966\_t001 |  | Atr-ERN19347 |  |  |  |  |  |  |  |  |  |  |
| 1 | Vvi-Vitvi16g00967\_t001 |  | \| \| |  |  |  |  |  |  |  |  |  |  |
| 1 | Vvi-Vitvi16g04344\_t001 |  | \| \| |  |  |  |  |  |  |  |  |  |  |
| 1 | Vvi-Vitvi16g00970\_t001 |  | \| \| |  |  |  |  |  |  |  |  |  |  |
| 1 | Vvi-Vitvi16g00971\_t001 |  | Atr-ERN19341 |  |  |  |  |  |  |  |  |  |  |
| 1 | Vvi-Vitvi16g00972\_t001 |  | Atr-ERN19338 |  |  |  |  |  |  |  |  |  |  |
| 1 | Vvi-Vitvi16g00973\_t001 |  | Atr-ERN19336 |  |  |  |  |  |  |  |  |  |  |
| 1 | Vvi-Vitvi16g00974\_t001 |  | \| \| |  |  |  |  |  |  |  |  |  |  |
| 1 | Vvi-Vitvi16g00975\_t001 |  | Atr-ERN19334 |  |  |  |  |  |  |  |  |  |  |
| 1 | Vvi-Vitvi16g00976\_t001 |  | \| \| |  |  |  |  |  |  |  |  |  |  |
| 1 | Vvi-Vitvi16g00977\_t001 |  | \| \| |  |  |  |  |  |  |  |  |  |  |
| 1 | Vvi-Vitvi16g00979\_t001 |  | \| \| |  |  |  |  |  |  |  |  |  |  |
| 1 | Vvi-Vitvi16g00982\_t001 |  | \| \| |  |  |  |  |  |  |  |  |  |  |
| 1 | Vvi-Vitvi16g00984\_t001 |  | Atr-ERN19319 |  |  |  |  |  |  |  |  |  |  |
| 0 | Vvi-Vitvi16g00985\_t001 |  |  |  |  |  |  |  |  |  |  |  |  |
| 0 | Vvi-Vitvi16g01485\_t001 |  |  |  |  |  |  |  |  |  |  |  |  |
| 0 | Vvi-Vitvi16g04345\_t001 |  |  |  |  |  |  |  |  |  |  |  |  |
| 0 | Vvi-Vitvi16g04346\_t001 |  |  |  |  |  |  |  |  |  |  |  |  |
| 0 | Vvi-Vitvi16g04347\_t001 |  |  |  |  |  |  |  |  |  |  |  |  |
| 0 | Vvi-Vitvi16g04348\_t001 |  |  |  |  |  |  |  |  |  |  |  |  |
| 0 | Vvi-Vitvi16g04349\_t001 |  |  |  |  |  |  |  |  |  |  |  |  |
| 0 | Vvi-Vitvi16g04350\_t001 |  |  |  |  |  |  |  |  |  |  |  |  |
| 0 | Vvi-Vitvi16g04351\_t001 |  |  |  |  |  |  |  |  |  |  |  |  |
| 0 | Vvi-Vitvi16g01473\_t001 |  |  |  |  |  |  |  |  |  |  |  |  |
| 0 | Vvi-Vitvi16g04352\_t001 |  |  |  |  |  |  |  |  |  |  |  |  |
| 0 | Vvi-Vitvi16g04353\_t001 |  |  |  |  |  |  |  |  |  |  |  |  |
| 0 | Vvi-Vitvi16g04354\_t001 |  |  |  |  |  |  |  |  |  |  |  |  |
| 0 | Vvi-Vitvi16g01000\_t001 |  |  |  |  |  |  |  |  |  |  |  |  |
| 0 | Vvi-Vitvi16g01871\_t003 |  |  |  |  |  |  |  |  |  |  |  |  |
| 0 | Vvi-Vitvi16g01001\_t001 |  |  |  |  |  |  |  |  |  |  |  |  |
| 1 | Vvi-Vitvi16g01872\_t001 |  | Atr-ERM95145 |  |  |  |  |  |  |  |  |  |  |
| 1 | Vvi-Vitvi16g01003\_t001 |  | \| \| |  |  |  |  |  |  |  |  |  |  |
| 1 | Vvi-Vitvi16g01004\_t001 |  | \| \| |  |  |  |  |  |  |  |  |  |  |
| 1 | Vvi-Vitvi16g01005\_t001 |  | \| \| |  |  |  |  |  |  |  |  |  |  |
| 1 | Vvi-Vitvi16g01006\_t001 |  | \| \| |  |  |  |  |  |  |  |  |  |  |
| 1 | Vvi-Vitvi16g01007\_t001 |  | \| \| |  |  |  |  |  |  |  |  |  |  |
| 1 | Vvi-Vitvi16g01008\_t001 |  | \| \| |  |  |  |  |  |  |  |  |  |  |
| 1 | Vvi-Vitvi16g01009\_t001 |  | Atr-ERM95127 |  |  |  |  |  |  |  |  |  |  |
| 1 | Vvi-Vitvi16g01011\_t001 |  | Atr-ERM95126 |  |  |  |  |  |  |  |  |  |  |
| 1 | Vvi-Vitvi16g01873\_t001 |  | \| \| |  |  |  |  |  |  |  |  |  |  |
| 1 | Vvi-Vitvi16g01012\_t001 |  | Atr-ERM95119 |  |  |  |  |  |  |  |  |  |  |
| 1 | Vvi-Vitvi16g01014\_t001 |  | \| \| |  |  |  |  |  |  |  |  |  |  |
| 1 | Vvi-Vitvi16g01015\_t001 |  | \| \| |  |  |  |  |  |  |  |  |  |  |
| 1 | Vvi-Vitvi16g01016\_t001 |  | \| \| |  |  |  |  |  |  |  |  |  |  |
| 1 | Vvi-Vitvi16g01017\_t001 |  | \| \| |  |  |  |  |  |  |  |  |  |  |
| 1 | Vvi-Vitvi16g01018\_t001 |  | Atr-ERM95109 |  |  |  |  |  |  |  |  |  |  |
| 1 | Vvi-Vitvi16g01019\_t001 |  | Atr-ERM95107 |  |  |  |  |  |  |  |  |  |  |
| 1 | Vvi-Vitvi16g01874\_t001 |  | Atr-ERM95105 |  |  |  |  |  |  |  |  |  |  |
| 1 | Vvi-Vitvi16g01021\_t001 |  | Atr-ERM95104 |  |  |  |  |  |  |  |  |  |  |
| 1 | Vvi-Vitvi16g01022\_t001 |  | Atr-ERM95103 |  |  |  |  |  |  |  |  |  |  |
| 1 | Vvi-Vitvi16g04355\_t001 |  | \| \| |  |  |  |  |  |  |  |  |  |  |
| 1 | Vvi-Vitvi16g01876\_t001 |  | \| \| |  |  |  |  |  |  |  |  |  |  |
| 1 | Vvi-Vitvi16g01023\_t001 |  | \| \| |  |  |  |  |  |  |  |  |  |  |
| 1 | Vvi-Vitvi16g01024\_t001 |  | \| \| |  |  |  |  |  |  |  |  |  |  |
| 1 | Vvi-Vitvi16g01877\_t001 |  | \| \| |  |  |  |  |  |  |  |  |  |  |
| 1 | Vvi-Vitvi16g01881\_t001 |  | \| \| |  |  |  |  |  |  |  |  |  |  |
| 1 | Vvi-Vitvi16g01026\_t001 |  | \| \| |  |  |  |  |  |  |  |  |  |  |
| 1 | Vvi-Vitvi16g01027\_t001 |  | Atr-ERM95097 |  |  |  |  |  |  |  |  |  |  |
| 1 | Vvi-Vitvi16g04356\_t001 |  | \| \| |  |  |  |  |  |  |  |  |  |  |
| 1 | Vvi-Vitvi16g01884\_t001 |  | \| \| |  |  |  |  |  |  |  |  |  |  |
| 1 | Vvi-Vitvi16g04357\_t001 |  | \| \| |  |  |  |  |  |  |  |  |  |  |
| 1 | Vvi-Vitvi16g04358\_t001 |  | \| \| |  |  |  |  |  |  |  |  |  |  |
| 1 | Vvi-Vitvi16g01029\_t001 |  | Atr-ERM95096 |  |  |  |  |  |  |  |  |  |  |
| 1 | Vvi-Vitvi16g01886\_t001 |  | \| \| |  |  |  |  |  |  |  |  |  |  |
| 1 | Vvi-Vitvi16g01031\_t001 |  | \| \| |  |  |  |  |  |  |  |  |  |  |
| 1 | Vvi-Vitvi16g04359\_t001 |  | \| \| |  |  |  |  |  |  |  |  |  |  |
| 1 | Vvi-Vitvi16g04360\_t001 |  | \| \| |  |  |  |  |  |  |  |  |  |  |
| 1 | Vvi-Vitvi16g04361\_t001 |  | \| \| |  |  |  |  |  |  |  |  |  |  |
| 1 | Vvi-Vitvi16g04362\_t001 |  | \| \| |  |  |  |  |  |  |  |  |  |  |
| 1 | Vvi-Vitvi16g04363\_t001 |  | \| \| |  |  |  |  |  |  |  |  |  |  |
| 1 | Vvi-Vitvi16g01891\_t001 |  | \| \| |  |  |  |  |  |  |  |  |  |  |
| 1 | Vvi-Vitvi16g01034\_t001 |  | \| \| |  |  |  |  |  |  |  |  |  |  |
| 1 | Vvi-Vitvi16g01894\_t001 |  | \| \| |  |  |  |  |  |  |  |  |  |  |
| 1 | Vvi-Vitvi16g01893\_t001 |  | \| \| |  |  |  |  |  |  |  |  |  |  |
| 1 | Vvi-Vitvi16g04364\_t001 |  | \| \| |  |  |  |  |  |  |  |  |  |  |
| 1 | Vvi-Vitvi16g01036\_t001 |  | Atr-ERM95094 |  |  |  |  |  |  |  |  |  |  |
| 1 | Vvi-Vitvi16g04365\_t001 |  | Atr-ERM95083 |  |  |  |  |  |  |  |  |  |  |
| 1 | Vvi-Vitvi16g01039\_t001 |  | \| \| |  |  |  |  |  |  |  |  |  |  |
| 1 | Vvi-Vitvi16g01041\_t002 |  | \| \| |  |  |  |  |  |  |  |  |  |  |
| 1 | Vvi-Vitvi16g01042\_t001 |  | \| \| |  |  |  |  |  |  |  |  |  |  |
| 1 | Vvi-Vitvi16g04366\_t001 |  | \| \| |  |  |  |  |  |  |  |  |  |  |
| 1 | Vvi-Vitvi16g04367\_t001 |  | \| \| |  |  |  |  |  |  |  |  |  |  |
| 1 | Vvi-Vitvi16g01045\_t001 |  | \| \| |  |  |  |  |  |  |  |  |  |  |
| 1 | Vvi-Vitvi16g01895\_t001 |  | \| \| |  |  |  |  |  |  |  |  |  |  |
| 1 | Vvi-Vitvi16g01896\_t001 |  | \| \| |  |  |  |  |  |  |  |  |  |  |
| 1 | Vvi-Vitvi16g01046\_t001 |  | \| \| |  |  |  |  |  |  |  |  |  |  |
| 1 | Vvi-Vitvi16g01897\_t002 |  | Atr-ERM95071 |  |  |  |  |  |  |  |  |  |  |
| 1 | Vvi-Vitvi16g01898\_t001 |  | \| \| |  |  |  |  |  |  |  |  |  |  |
| 1 | Vvi-Vitvi16g01899\_t001 |  | \| \| |  |  |  |  |  |  |  |  |  |  |
| 1 | Vvi-Vitvi16g01900\_t001 |  | \| \| |  |  |  |  |  |  |  |  |  |  |
| 1 | Vvi-Vitvi16g04368\_t001 |  | \| \| |  |  |  |  |  |  |  |  |  |  |
| 1 | Vvi-Vitvi16g04369\_t001 |  | \| \| |  |  |  |  |  |  |  |  |  |  |
| 1 | Vvi-Vitvi16g04370\_t001 |  | \| \| |  |  |  |  |  |  |  |  |  |  |
| 1 | Vvi-Vitvi16g04371\_t001 |  | \| \| |  |  |  |  |  |  |  |  |  |  |
| 1 | Vvi-Vitvi16g04372\_t001 |  | \| \| |  |  |  |  |  |  |  |  |  |  |
| 1 | Vvi-Vitvi16g04373\_t001 |  | \| \| |  |  |  |  |  |  |  |  |  |  |
| 1 | Vvi-Vitvi16g01051\_t001 |  | Atr-ERM95061 |  |  |  |  |  |  |  |  |  |  |
| 1 | Vvi-Vitvi16g04374\_t001 |  | \| \| |  |  |  |  |  |  |  |  |  |  |
| 1 | Vvi-Vitvi16g04375\_t001 |  | \| \| |  |  |  |  |  |  |  |  |  |  |
| 1 | Vvi-Vitvi16g01052\_t001 |  | Atr-ERM95057 |  |  |  |  |  |  |  |  |  |  |
| 1 | Vvi-Vitvi16g01053\_t001 |  | Atr-ERM95044 |  |  |  |  |  |  |  |  |  |  |
| 1 | Vvi-Vitvi16g01054\_t002 |  | \| \| |  |  |  |  |  |  |  |  |  |  |
| 1 | Vvi-Vitvi16g01055\_t002 |  | \| \| |  |  |  |  |  |  |  |  |  |  |
| 1 | Vvi-Vitvi16g01056\_t001 |  | \| \| |  |  |  |  |  |  |  |  |  |  |
| 1 | Vvi-Vitvi16g01058\_t001 |  | Atr-ERM95036 |  |  |  |  |  |  |  |  |  |  |
| 1 | Vvi-Vitvi16g01060\_t001 |  | \| \| |  |  |  |  |  |  |  |  |  |  |
| 1 | Vvi-Vitvi16g01903\_t001 |  | \| \| |  |  |  |  |  |  |  |  |  |  |
| 1 | Vvi-Vitvi16g01061\_t001 |  | \| \| |  |  |  |  |  |  |  |  |  |  |
| 1 | Vvi-Vitvi16g01062\_t001 |  | \| \| |  |  |  |  |  |  |  |  |  |  |
| 1 | Vvi-Vitvi16g01063\_t002 |  | Atr-ERM95019 |  |  |  |  |  |  |  |  |  |  |
| 1 | Vvi-Vitvi16g01065\_t001 |  | Atr-ERM95010 |  |  |  |  |  |  |  |  |  |  |
| 1 | Vvi-Vitvi16g01066\_t001 |  | Atr-ERM95008 |  |  |  |  |  |  |  |  |  |  |
| 1 | Vvi-Vitvi16g01067\_t001 |  | Atr-ERM95002 |  |  |  |  |  |  |  |  |  |  |
| 1 | Vvi-Vitvi16g01904\_t001 |  | Atr-ERM94999 |  |  |  |  |  |  |  |  |  |  |
| 1 | Vvi-Vitvi16g01068\_t002 |  | Atr-ERM94997 |  |  |  |  |  |  |  |  |  |  |
| 1 | Vvi-Vitvi16g04376\_t001 |  | Atr-ERM94993 |  |  |  |  |  |  |  |  |  |  |
| 1 | Vvi-Vitvi16g01907\_t001 |  | \| \| |  |  |  |  |  |  |  |  |  |  |
| 1 | Vvi-Vitvi16g01908\_t001 |  | \| \| |  |  |  |  |  |  |  |  |  |  |
| 1 | Vvi-Vitvi16g01070\_t001 |  | \| \| |  |  |  |  |  |  |  |  |  |  |
| 1 | Vvi-Vitvi16g01071\_t001 |  | Atr-ERM94991 |  |  |  |  |  |  |  |  |  |  |
| 1 | Vvi-Vitvi16g01073\_t001 |  | Atr-ERM94988 |  |  |  |  |  |  |  |  |  |  |
| 1 | Vvi-Vitvi16g01074\_t001 |  | Atr-ERM94986 |  |  |  |  |  |  |  |  |  |  |
| 1 | Vvi-Vitvi16g01909\_t001 |  | Atr-ERM94984 |  |  |  |  |  |  |  |  |  |  |
| 1 | Vvi-Vitvi16g01075\_t001 |  | \| \| |  |  |  |  |  |  |  |  |  |  |
| 1 | Vvi-Vitvi16g01076\_t001 |  | \| \| |  |  |  |  |  |  |  |  |  |  |
| 1 | Vvi-Vitvi16g01078\_t001 |  | Atr-ERM94982 |  |  |  |  |  |  |  |  |  |  |
| 1 | Vvi-Vitvi16g01079\_t001 |  | Atr-ERM94981 |  |  |  |  |  |  |  |  |  |  |
| 1 | Vvi-Vitvi16g01910\_t001 |  | \| \| |  |  |  |  |  |  |  |  |  |  |
| 1 | Vvi-Vitvi16g01080\_t001 |  | Atr-ERM94972 |  |  |  |  |  |  |  |  |  |  |
| 1 | Vvi-Vitvi16g01081\_t001 |  | \| \| |  |  |  |  |  |  |  |  |  |  |
| 1 | Vvi-Vitvi16g01082\_t001 |  | \| \| |  |  |  |  |  |  |  |  |  |  |
| 1 | Vvi-Vitvi16g01083\_t001 |  | Atr-ERM94968 |  |  |  |  |  |  |  |  |  |  |
| 1 | Vvi-Vitvi16g01911\_t001 |  | \| \| |  |  |  |  |  |  |  |  |  |  |
| 1 | Vvi-Vitvi16g01912\_t001 |  | Atr-ERM94951 |  |  |  |  |  |  |  |  |  |  |
| 1 | Vvi-Vitvi16g04377\_t001 |  | Atr-ERM94950 |  |  |  |  |  |  |  |  |  |  |
| 0 | Vvi-Vitvi16g01915\_t003 |  |  |  |  |  |  |  |  |  |  |  |  |
| 0 | Vvi-Vitvi16g01086\_t001 |  |  |  |  |  |  |  |  |  |  |  |  |
| 0 | Vvi-Vitvi16g01087\_t001 |  |  |  |  |  |  |  |  |  |  |  |  |
| 0 | Vvi-Vitvi16g01089\_t001 |  |  |  |  |  |  |  |  |  |  |  |  |
| 1 | Vvi-Vitvi16g01090\_t001 |  | Atr-ERM96456 |  |  |  |  |  |  |  |  |  |  |
| 1 | Vvi-Vitvi16g01091\_t001 |  | \| \| |  |  |  |  |  |  |  |  |  |  |
| 1 | Vvi-Vitvi16g01093\_t001 |  | \| \| |  |  |  |  |  |  |  |  |  |  |
| 1 | Vvi-Vitvi16g01916\_t001 |  | Atr-ERM96460 |  |  |  |  |  |  |  |  |  |  |
| 1 | Vvi-Vitvi16g04378\_t001 |  | \| \| |  |  |  |  |  |  |  |  |  |  |
| 1 | Vvi-Vitvi16g01094\_t001 |  | \| \| |  |  |  |  |  |  |  |  |  |  |
| 1 | Vvi-Vitvi16g01095\_t001 |  | Atr-ERM96463 |  |  |  |  |  |  |  |  |  |  |
| 1 | Vvi-Vitvi16g01096\_t001 |  | Atr-ERM96475 |  |  |  |  |  |  |  |  |  |  |
| 1 | Vvi-Vitvi16g01097\_t001 |  | Atr-ERM96489 |  |  |  |  |  |  |  |  |  |  |
| 1 | Vvi-Vitvi16g01098\_t001 |  | \| \| |  |  |  |  |  |  |  |  |  |  |
| 1 | Vvi-Vitvi16g01099\_t001 |  | Atr-ERM96490 |  |  |  |  |  |  |  |  |  |  |
| 1 | Vvi-Vitvi16g01919\_t001 |  | \| \| |  |  |  |  |  |  |  |  |  |  |
| 1 | Vvi-Vitvi16g01920\_t001 |  | \| \| |  |  |  |  |  |  |  |  |  |  |
| 1 | Vvi-Vitvi16g01921\_t001 |  | \| \| |  |  |  |  |  |  |  |  |  |  |
| 1 | Vvi-Vitvi16g01922\_t001 |  | \| \| |  |  |  |  |  |  |  |  |  |  |
| 1 | Vvi-Vitvi16g01100\_t001 |  | \| \| |  |  |  |  |  |  |  |  |  |  |
| 1 | Vvi-Vitvi16g01101\_t001 |  | \| \| |  |  |  |  |  |  |  |  |  |  |
| 1 | Vvi-Vitvi16g01102\_t001 |  | \| \| |  |  |  |  |  |  |  |  |  |  |
| 1 | Vvi-Vitvi16g01103\_t001 |  | \| \| |  |  |  |  |  |  |  |  |  |  |
| 1 | Vvi-Vitvi16g01104\_t001 |  | \| \| |  |  |  |  |  |  |  |  |  |  |
| 1 | Vvi-Vitvi16g01105\_t001 |  | Atr-ERM96502 |  |  |  |  |  |  |  |  |  |  |
| 1 | Vvi-Vitvi16g01106\_t001 |  | Atr-ERM96503 |  |  |  |  |  |  |  |  |  |  |
| 1 | Vvi-Vitvi16g04379\_t001 |  | \| \| |  |  |  |  |  |  |  |  |  |  |
| 1 | Vvi-Vitvi16g01923\_t001 |  | \| \| |  |  |  |  |  |  |  |  |  |  |
| 1 | Vvi-Vitvi16g01924\_t001 |  | \| \| |  |  |  |  |  |  |  |  |  |  |
| 1 | Vvi-Vitvi16g01926\_t001 |  | \| \| |  |  |  |  |  |  |  |  |  |  |
| 1 | Vvi-Vitvi16g01112\_t001 |  | \| \| |  |  |  |  |  |  |  |  |  |  |
| 1 | Vvi-Vitvi16g01113\_t001 |  | \| \| |  |  |  |  |  |  |  |  |  |  |
| 1 | Vvi-Vitvi16g01928\_t001 |  | \| \| |  |  |  |  |  |  |  |  |  |  |
| 1 | Vvi-Vitvi16g01929\_t001 |  | \| \| |  |  |  |  |  |  |  |  |  |  |
| 1 | Vvi-Vitvi16g04380\_t001 |  | \| \| |  |  |  |  |  |  |  |  |  |  |
| 1 | Vvi-Vitvi16g04381\_t001 |  | \| \| |  |  |  |  |  |  |  |  |  |  |
| 1 | Vvi-Vitvi16g01930\_t001 |  | \| \| |  |  |  |  |  |  |  |  |  |  |
| 1 | Vvi-Vitvi16g04382\_t001 |  | \| \| |  |  |  |  |  |  |  |  |  |  |
| 1 | Vvi-Vitvi16g01118\_t001 |  | \| \| |  |  |  |  |  |  |  |  |  |  |
| 1 | Vvi-Vitvi16g04383\_t001 |  | \| \| |  |  |  |  |  |  |  |  |  |  |
| 1 | Vvi-Vitvi16g01119\_t001 |  | \| \| |  |  |  |  |  |  |  |  |  |  |
| 1 | Vvi-Vitvi16g04384\_t001 |  | \| \| |  |  |  |  |  |  |  |  |  |  |
| 1 | Vvi-Vitvi16g04385\_t001 |  | \| \| |  |  |  |  |  |  |  |  |  |  |
| 1 | Vvi-Vitvi16g04386\_t001 |  | \| \| |  |  |  |  |  |  |  |  |  |  |
| 1 | Vvi-Vitvi16g01931\_t001 |  | \| \| |  |  |  |  |  |  |  |  |  |  |
| 1 | Vvi-Vitvi16g04387\_t001 |  | \| \| |  |  |  |  |  |  |  |  |  |  |
| 1 | Vvi-Vitvi16g01932\_t001 |  | \| \| |  |  |  |  |  |  |  |  |  |  |
| 1 | Vvi-Vitvi16g01935\_t001 |  | \| \| |  |  |  |  |  |  |  |  |  |  |
| 1 | Vvi-Vitvi16g01936\_t001 |  | \| \| |  |  |  |  |  |  |  |  |  |  |
| 1 | Vvi-Vitvi16g01126\_t001 |  | \| \| |  |  |  |  |  |  |  |  |  |  |
| 1 | Vvi-Vitvi16g01127\_t001 |  | Atr-ERM96506 |  |  |  |  |  |  |  |  |  |  |
| 0 | Vvi-Vitvi16g01128\_t001 |  |  |  |  |  |  |  |  |  |  |  |  |
| 0 | Vvi-Vitvi16g01129\_t001 |  |  |  |  |  |  |  |  |  |  |  |  |
| 0 | Vvi-Vitvi16g01130\_t001 |  |  |  |  |  |  |  |  |  |  |  |  |
| 0 | Vvi-Vitvi16g01131\_t001 |  |  |  |  |  |  |  |  |  |  |  |  |
| 0 | Vvi-Vitvi16g01132\_t001 |  |  |  |  |  |  |  |  |  |  |  |  |
| 0 | Vvi-Vitvi16g01133\_t001 |  |  |  |  |  |  |  |  |  |  |  |  |
| 0 | Vvi-Vitvi16g01134\_t001 |  |  |  |  |  |  |  |  |  |  |  |  |
| 0 | Vvi-Vitvi16g01135\_t001 |  |  |  |  |  |  |  |  |  |  |  |  |
| 0 | Vvi-Vitvi16g01136\_t003 |  |  |  |  |  |  |  |  |  |  |  |  |
| 0 | Vvi-Vitvi16g01937\_t001 |  |  |  |  |  |  |  |  |  |  |  |  |
| 0 | Vvi-Vitvi16g04388\_t001 |  |  |  |  |  |  |  |  |  |  |  |  |
| 0 | Vvi-Vitvi16g01938\_t001 |  |  |  |  |  |  |  |  |  |  |  |  |
| 0 | Vvi-Vitvi16g04389\_t001 |  |  |  |  |  |  |  |  |  |  |  |  |
| 0 | Vvi-Vitvi16g01141\_t001 |  |  |  |  |  |  |  |  |  |  |  |  |
| 0 | Vvi-Vitvi16g01942\_t004 |  |  |  |  |  |  |  |  |  |  |  |  |
| 0 | Vvi-Vitvi16g04390\_t001 |  |  |  |  |  |  |  |  |  |  |  |  |
| 0 | Vvi-Vitvi16g01940\_t001 |  |  |  |  |  |  |  |  |  |  |  |  |
| 0 | Vvi-Vitvi16g01941\_t001 |  |  |  |  |  |  |  |  |  |  |  |  |
| 0 | Vvi-Vitvi16g01943\_t001 |  |  |  |  |  |  |  |  |  |  |  |  |
| 0 | Vvi-Vitvi16g01142\_t001 |  |  |  |  |  |  |  |  |  |  |  |  |
| 0 | Vvi-Vitvi16g01946\_t001 |  |  |  |  |  |  |  |  |  |  |  |  |
| 0 | Vvi-Vitvi16g01144\_t001 |  |  |  |  |  |  |  |  |  |  |  |  |
| 0 | Vvi-Vitvi16g01146\_t001 |  |  |  |  |  |  |  |  |  |  |  |  |
| 0 | Vvi-Vitvi16g01149\_t001 |  |  |  |  |  |  |  |  |  |  |  |  |
| 0 | Vvi-Vitvi16g04391\_t001 |  |  |  |  |  |  |  |  |  |  |  |  |
| 0 | Vvi-Vitvi16g01150\_t001 |  |  |  |  |  |  |  |  |  |  |  |  |
| 0 | Vvi-Vitvi16g01948\_t001 |  |  |  |  |  |  |  |  |  |  |  |  |
| 0 | Vvi-Vitvi16g01151\_t001 |  |  |  |  |  |  |  |  |  |  |  |  |
| 0 | Vvi-Vitvi16g04392\_t001 |  |  |  |  |  |  |  |  |  |  |  |  |
| 0 | Vvi-Vitvi16g01152\_t001 |  |  |  |  |  |  |  |  |  |  |  |  |
| 0 | Vvi-Vitvi16g04393\_t001 |  |  |  |  |  |  |  |  |  |  |  |  |
| 0 | Vvi-Vitvi16g04394\_t001 |  |  |  |  |  |  |  |  |  |  |  |  |
| 0 | Vvi-Vitvi16g01950\_t001 |  |  |  |  |  |  |  |  |  |  |  |  |
| 0 | Vvi-Vitvi16g04395\_t001 |  |  |  |  |  |  |  |  |  |  |  |  |
| 0 | Vvi-Vitvi16g01951\_t001 |  |  |  |  |  |  |  |  |  |  |  |  |
| 0 | Vvi-Vitvi16g01952\_t001 |  |  |  |  |  |  |  |  |  |  |  |  |
| 0 | Vvi-Vitvi16g01159\_t001 |  |  |  |  |  |  |  |  |  |  |  |  |
| 0 | Vvi-Vitvi16g01953\_t001 |  |  |  |  |  |  |  |  |  |  |  |  |
| 0 | Vvi-Vitvi16g04396\_t001 |  |  |  |  |  |  |  |  |  |  |  |  |
| 0 | Vvi-Vitvi16g01954\_t001 |  |  |  |  |  |  |  |  |  |  |  |  |
| 0 | Vvi-Vitvi16g04397\_t001 |  |  |  |  |  |  |  |  |  |  |  |  |
| 0 | Vvi-Vitvi16g01161\_t001 |  |  |  |  |  |  |  |  |  |  |  |  |
| 0 | Vvi-Vitvi16g01162\_t002 |  |  |  |  |  |  |  |  |  |  |  |  |
| 0 | Vvi-Vitvi16g01957\_t001 |  |  |  |  |  |  |  |  |  |  |  |  |
| 0 | Vvi-Vitvi16g01958\_t002 |  |  |  |  |  |  |  |  |  |  |  |  |
| 0 | Vvi-Vitvi16g01163\_t001 |  |  |  |  |  |  |  |  |  |  |  |  |
| 0 | Vvi-Vitvi16g01164\_t001 |  |  |  |  |  |  |  |  |  |  |  |  |
| 0 | Vvi-Vitvi16g04398\_t001 |  |  |  |  |  |  |  |  |  |  |  |  |
| 0 | Vvi-Vitvi16g01959\_t001 |  |  |  |  |  |  |  |  |  |  |  |  |
| 0 | Vvi-Vitvi16g04399\_t001 |  |  |  |  |  |  |  |  |  |  |  |  |
| 0 | Vvi-Vitvi16g01166\_t001 |  |  |  |  |  |  |  |  |  |  |  |  |
| 0 | Vvi-Vitvi16g01167\_t001 |  |  |  |  |  |  |  |  |  |  |  |  |
| 0 | Vvi-Vitvi16g01960\_t001 |  |  |  |  |  |  |  |  |  |  |  |  |
| 0 | Vvi-Vitvi16g04400\_t001 |  |  |  |  |  |  |  |  |  |  |  |  |
| 0 | Vvi-Vitvi16g04401\_t001 |  |  |  |  |  |  |  |  |  |  |  |  |
| 0 | Vvi-Vitvi16g04402\_t001 |  |  |  |  |  |  |  |  |  |  |  |  |
| 0 | Vvi-Vitvi16g01963\_t001 |  |  |  |  |  |  |  |  |  |  |  |  |
| 0 | Vvi-Vitvi16g04403\_t001 |  |  |  |  |  |  |  |  |  |  |  |  |
| 0 | Vvi-Vitvi16g01964\_t001 |  |  |  |  |  |  |  |  |  |  |  |  |
| 0 | Vvi-Vitvi16g04404\_t001 |  |  |  |  |  |  |  |  |  |  |  |  |
| 0 | Vvi-Vitvi16g04405\_t001 |  |  |  |  |  |  |  |  |  |  |  |  |
| 0 | Vvi-Vitvi16g01965\_t001 |  |  |  |  |  |  |  |  |  |  |  |  |
| 0 | Vvi-Vitvi16g01966\_t001 |  |  |  |  |  |  |  |  |  |  |  |  |
| 0 | Vvi-Vitvi16g01967\_t001 |  |  |  |  |  |  |  |  |  |  |  |  |
| 0 | Vvi-Vitvi16g04406\_t001 |  |  |  |  |  |  |  |  |  |  |  |  |
| 0 | Vvi-Vitvi16g04407\_t001 |  |  |  |  |  |  |  |  |  |  |  |  |
| 0 | Vvi-Vitvi16g04408\_t001 |  |  |  |  |  |  |  |  |  |  |  |  |
| 0 | Vvi-Vitvi16g01968\_t001 |  |  |  |  |  |  |  |  |  |  |  |  |
| 0 | Vvi-Vitvi16g01171\_t001 |  |  |  |  |  |  |  |  |  |  |  |  |
| 0 | Vvi-Vitvi16g01969\_t001 |  |  |  |  |  |  |  |  |  |  |  |  |
| 0 | Vvi-Vitvi16g04409\_t001 |  |  |  |  |  |  |  |  |  |  |  |  |
| 0 | Vvi-Vitvi16g04410\_t001 |  |  |  |  |  |  |  |  |  |  |  |  |
| 0 | Vvi-Vitvi16g04411\_t001 |  |  |  |  |  |  |  |  |  |  |  |  |
| 0 | Vvi-Vitvi16g04412\_t001 |  |  |  |  |  |  |  |  |  |  |  |  |
| 0 | Vvi-Vitvi16g01973\_t001 |  |  |  |  |  |  |  |  |  |  |  |  |
| 0 | Vvi-Vitvi16g04413\_t001 |  |  |  |  |  |  |  |  |  |  |  |  |
| 0 | Vvi-Vitvi16g01970\_t001 |  |  |  |  |  |  |  |  |  |  |  |  |
| 0 | Vvi-Vitvi16g04414\_t001 |  |  |  |  |  |  |  |  |  |  |  |  |
| 0 | Vvi-Vitvi16g01173\_t001 |  |  |  |  |  |  |  |  |  |  |  |  |
| 0 | Vvi-Vitvi16g04415\_t001 |  |  |  |  |  |  |  |  |  |  |  |  |
| 0 | Vvi-Vitvi16g04416\_t001 |  |  |  |  |  |  |  |  |  |  |  |  |
| 0 | Vvi-Vitvi16g01175\_t001 |  |  |  |  |  |  |  |  |  |  |  |  |
| 0 | Vvi-Vitvi16g04417\_t001 |  |  |  |  |  |  |  |  |  |  |  |  |
| 0 | Vvi-Vitvi16g04418\_t001 |  |  |  |  |  |  |  |  |  |  |  |  |
| 0 | Vvi-Vitvi16g04419\_t001 |  |  |  |  |  |  |  |  |  |  |  |  |
| 0 | Vvi-Vitvi16g04420\_t001 |  |  |  |  |  |  |  |  |  |  |  |  |
| 0 | Vvi-Vitvi16g04421\_t001 |  |  |  |  |  |  |  |  |  |  |  |  |
| 0 | Vvi-Vitvi16g04422\_t001 |  |  |  |  |  |  |  |  |  |  |  |  |
| 0 | Vvi-Vitvi16g01181\_t001 |  |  |  |  |  |  |  |  |  |  |  |  |
| 0 | Vvi-Vitvi16g01182\_t001 |  |  |  |  |  |  |  |  |  |  |  |  |
| 0 | Vvi-Vitvi16g01183\_t001 |  |  |  |  |  |  |  |  |  |  |  |  |
| 0 | Vvi-Vitvi16g04423\_t001 |  |  |  |  |  |  |  |  |  |  |  |  |
| 0 | Vvi-Vitvi16g01978\_t001 |  |  |  |  |  |  |  |  |  |  |  |  |
| 0 | Vvi-Vitvi16g01979\_t001 |  |  |  |  |  |  |  |  |  |  |  |  |
| 0 | Vvi-Vitvi16g04424\_t001 |  |  |  |  |  |  |  |  |  |  |  |  |
| 0 | Vvi-Vitvi16g04425\_t001 |  |  |  |  |  |  |  |  |  |  |  |  |
| 0 | Vvi-Vitvi16g01980\_t001 |  |  |  |  |  |  |  |  |  |  |  |  |
| 0 | Vvi-Vitvi16g01981\_t001 |  |  |  |  |  |  |  |  |  |  |  |  |
| 0 | Vvi-Vitvi16g04426\_t001 |  |  |  |  |  |  |  |  |  |  |  |  |
| 0 | Vvi-Vitvi16g01184\_t001 |  |  |  |  |  |  |  |  |  |  |  |  |
| 0 | Vvi-Vitvi16g01185\_t001 |  |  |  |  |  |  |  |  |  |  |  |  |
| 0 | Vvi-Vitvi16g01187\_t001 |  |  |  |  |  |  |  |  |  |  |  |  |
| 0 | Vvi-Vitvi16g01188\_t001 |  |  |  |  |  |  |  |  |  |  |  |  |
| 0 | Vvi-Vitvi16g01190\_t001 |  |  |  |  |  |  |  |  |  |  |  |  |
| 0 | Vvi-Vitvi16g01982\_t001 |  |  |  |  |  |  |  |  |  |  |  |  |
| 0 | Vvi-Vitvi16g01191\_t001 |  |  |  |  |  |  |  |  |  |  |  |  |
| 0 | Vvi-Vitvi16g01192\_t001 |  |  |  |  |  |  |  |  |  |  |  |  |
| 0 | Vvi-Vitvi16g01193\_t001 |  |  |  |  |  |  |  |  |  |  |  |  |
| 0 | Vvi-Vitvi16g01194\_t003 |  |  |  |  |  |  |  |  |  |  |  |  |
| 0 | Vvi-Vitvi16g01195\_t002 |  |  |  |  |  |  |  |  |  |  |  |  |
| 0 | Vvi-Vitvi16g04427\_t001 |  |  |  |  |  |  |  |  |  |  |  |  |
| 0 | Vvi-Vitvi16g04428\_t001 |  |  |  |  |  |  |  |  |  |  |  |  |
| 0 | Vvi-Vitvi16g01197\_t001 |  |  |  |  |  |  |  |  |  |  |  |  |
| 0 | Vvi-Vitvi16g01198\_t001 |  |  |  |  |  |  |  |  |  |  |  |  |
| 0 | Vvi-Vitvi16g01199\_t001 |  |  |  |  |  |  |  |  |  |  |  |  |
| 0 | Vvi-Vitvi16g04429\_t001 |  |  |  |  |  |  |  |  |  |  |  |  |
| 0 | Vvi-Vitvi16g01200\_t001 |  |  |  |  |  |  |  |  |  |  |  |  |
| 0 | Vvi-Vitvi16g01983\_t001 |  |  |  |  |  |  |  |  |  |  |  |  |
| 0 | Vvi-Vitvi16g01201\_t001 |  |  |  |  |  |  |  |  |  |  |  |  |
| 0 | Vvi-Vitvi16g04430\_t001 |  |  |  |  |  |  |  |  |  |  |  |  |
| 0 | Vvi-Vitvi16g01203\_t002 |  |  |  |  |  |  |  |  |  |  |  |  |
| 0 | Vvi-Vitvi16g01204\_t001 |  |  |  |  |  |  |  |  |  |  |  |  |
| 0 | Vvi-Vitvi16g01205\_t001 |  |  |  |  |  |  |  |  |  |  |  |  |
| 0 | Vvi-Vitvi16g01206\_t001 |  |  |  |  |  |  |  |  |  |  |  |  |
| 0 | Vvi-Vitvi16g01207\_t002 |  |  |  |  |  |  |  |  |  |  |  |  |
| 0 | Vvi-Vitvi16g01208\_t001 |  |  |  |  |  |  |  |  |  |  |  |  |
| 0 | Vvi-Vitvi16g01209\_t001 |  |  |  |  |  |  |  |  |  |  |  |  |
| 0 | Vvi-Vitvi16g04431\_t001 |  |  |  |  |  |  |  |  |  |  |  |  |
| 0 | Vvi-Vitvi16g04432\_t001 |  |  |  |  |  |  |  |  |  |  |  |  |
| 0 | Vvi-Vitvi16g01210\_t005 |  |  |  |  |  |  |  |  |  |  |  |  |
| 0 | Vvi-Vitvi16g01211\_t001 |  |  |  |  |  |  |  |  |  |  |  |  |
| 0 | Vvi-Vitvi16g01212\_t001 |  |  |  |  |  |  |  |  |  |  |  |  |
| 0 | Vvi-Vitvi16g01213\_t001 |  |  |  |  |  |  |  |  |  |  |  |  |
| 0 | Vvi-Vitvi16g04433\_t001 |  |  |  |  |  |  |  |  |  |  |  |  |
| 0 | Vvi-Vitvi16g01214\_t001 |  |  |  |  |  |  |  |  |  |  |  |  |
| 0 | Vvi-Vitvi16g01215\_t001 |  |  |  |  |  |  |  |  |  |  |  |  |
| 0 | Vvi-Vitvi16g01984\_t001 |  |  |  |  |  |  |  |  |  |  |  |  |
| 0 | Vvi-Vitvi16g01217\_t001 |  |  |  |  |  |  |  |  |  |  |  |  |
| 0 | Vvi-Vitvi16g01218\_t001 |  |  |  |  |  |  |  |  |  |  |  |  |
| 0 | Vvi-Vitvi16g04434\_t001 |  |  |  |  |  |  |  |  |  |  |  |  |
| 0 | Vvi-Vitvi16g01220\_t001 |  |  |  |  |  |  |  |  |  |  |  |  |
| 0 | Vvi-Vitvi16g01221\_t002 |  |  |  |  |  |  |  |  |  |  |  |  |
| 0 | Vvi-Vitvi16g01222\_t001 |  |  |  |  |  |  |  |  |  |  |  |  |
| 0 | Vvi-Vitvi16g01223\_t001 |  |  |  |  |  |  |  |  |  |  |  |  |
| 0 | Vvi-Vitvi16g01225\_t001 |  |  |  |  |  |  |  |  |  |  |  |  |
| 0 | Vvi-Vitvi16g04435\_t001 |  |  |  |  |  |  |  |  |  |  |  |  |
| 0 | Vvi-Vitvi16g04436\_t001 |  |  |  |  |  |  |  |  |  |  |  |  |
| 0 | Vvi-Vitvi16g01226\_t001 |  |  |  |  |  |  |  |  |  |  |  |  |
| 0 | Vvi-Vitvi16g01229\_t001 |  |  |  |  |  |  |  |  |  |  |  |  |
| 0 | Vvi-Vitvi16g01230\_t001 |  |  |  |  |  |  |  |  |  |  |  |  |
| 0 | Vvi-Vitvi16g04437\_t001 |  |  |  |  |  |  |  |  |  |  |  |  |
| 0 | Vvi-Vitvi16g01233\_t002 |  |  |  |  |  |  |  |  |  |  |  |  |
| 0 | Vvi-Vitvi16g01985\_t001 |  |  |  |  |  |  |  |  |  |  |  |  |
| 0 | Vvi-Vitvi16g04438\_t001 |  |  |  |  |  |  |  |  |  |  |  |  |
| 0 | Vvi-Vitvi16g04439\_t001 |  |  |  |  |  |  |  |  |  |  |  |  |
| 0 | Vvi-Vitvi16g04440\_t001 |  |  |  |  |  |  |  |  |  |  |  |  |
| 0 | Vvi-Vitvi16g04441\_t001 |  |  |  |  |  |  |  |  |  |  |  |  |
| 0 | Vvi-Vitvi16g04442\_t001 |  |  |  |  |  |  |  |  |  |  |  |  |
| 0 | Vvi-Vitvi16g04443\_t001 |  |  |  |  |  |  |  |  |  |  |  |  |
| 0 | Vvi-Vitvi16g04444\_t001 |  |  |  |  |  |  |  |  |  |  |  |  |
| 0 | Vvi-Vitvi16g02122\_t001 |  |  |  |  |  |  |  |  |  |  |  |  |
| 0 | Vvi-Vitvi16g04445\_t001 |  |  |  |  |  |  |  |  |  |  |  |  |
| 0 | Vvi-Vitvi16g04446\_t001 |  |  |  |  |  |  |  |  |  |  |  |  |
| 0 | Vvi-Vitvi16g04447\_t001 |  |  |  |  |  |  |  |  |  |  |  |  |
| 0 | Vvi-Vitvi16g04448\_t001 |  |  |  |  |  |  |  |  |  |  |  |  |
| 0 | Vvi-Vitvi16g04449\_t001 |  |  |  |  |  |  |  |  |  |  |  |  |
| 0 | Vvi-Vitvi16g04450\_t001 |  |  |  |  |  |  |  |  |  |  |  |  |
| 0 | Vvi-Vitvi16g04451\_t001 |  |  |  |  |  |  |  |  |  |  |  |  |
| 0 | Vvi-Vitvi16g01243\_t001 |  |  |  |  |  |  |  |  |  |  |  |  |
| 0 | Vvi-Vitvi16g04452\_t001 |  |  |  |  |  |  |  |  |  |  |  |  |
| 0 | Vvi-Vitvi16g04453\_t001 |  |  |  |  |  |  |  |  |  |  |  |  |
| 0 | Vvi-Vitvi16g04454\_t001 |  |  |  |  |  |  |  |  |  |  |  |  |
| 0 | Vvi-Vitvi16g01236\_t001 |  |  |  |  |  |  |  |  |  |  |  |  |
| 0 | Vvi-Vitvi16g04455\_t001 |  |  |  |  |  |  |  |  |  |  |  |  |
| 0 | Vvi-Vitvi16g04456\_t001 |  |  |  |  |  |  |  |  |  |  |  |  |
| 0 | Vvi-Vitvi16g04457\_t001 |  |  |  |  |  |  |  |  |  |  |  |  |
| 0 | Vvi-Vitvi16g01246\_t001 |  |  |  |  |  |  |  |  |  |  |  |  |
| 0 | Vvi-Vitvi16g04458\_t001 |  |  |  |  |  |  |  |  |  |  |  |  |
| 0 | Vvi-Vitvi16g02012\_t001 |  |  |  |  |  |  |  |  |  |  |  |  |
| 0 | Vvi-Vitvi16g04459\_t001 |  |  |  |  |  |  |  |  |  |  |  |  |
| 0 | Vvi-Vitvi16g04460\_t001 |  |  |  |  |  |  |  |  |  |  |  |  |
| 0 | Vvi-Vitvi16g04461\_t001 |  |  |  |  |  |  |  |  |  |  |  |  |
| 0 | Vvi-Vitvi16g04462\_t001 |  |  |  |  |  |  |  |  |  |  |  |  |
| 0 | Vvi-Vitvi16g04463\_t001 |  |  |  |  |  |  |  |  |  |  |  |  |
| 0 | Vvi-Vitvi16g04464\_t001 |  |  |  |  |  |  |  |  |  |  |  |  |
| 0 | Vvi-Vitvi16g04465\_t001 |  |  |  |  |  |  |  |  |  |  |  |  |
| 0 | Vvi-Vitvi16g02116\_t001 |  |  |  |  |  |  |  |  |  |  |  |  |
| 0 | Vvi-Vitvi16g04466\_t001 |  |  |  |  |  |  |  |  |  |  |  |  |
| 0 | Vvi-Vitvi16g04467\_t001 |  |  |  |  |  |  |  |  |  |  |  |  |
| 0 | Vvi-Vitvi16g04468\_t001 |  |  |  |  |  |  |  |  |  |  |  |  |
| 0 | Vvi-Vitvi16g04469\_t001 |  |  |  |  |  |  |  |  |  |  |  |  |
| 0 | Vvi-Vitvi16g01266\_t001 |  |  |  |  |  |  |  |  |  |  |  |  |
| 0 | Vvi-Vitvi16g04470\_t001 |  |  |  |  |  |  |  |  |  |  |  |  |
| 0 | Vvi-Vitvi16g02035\_t001 |  |  |  |  |  |  |  |  |  |  |  |  |
| 0 | Vvi-Vitvi16g04471\_t001 |  |  |  |  |  |  |  |  |  |  |  |  |
| 0 | Vvi-Vitvi16g04472\_t001 |  |  |  |  |  |  |  |  |  |  |  |  |
| 0 | Vvi-Vitvi16g04473\_t001 |  |  |  |  |  |  |  |  |  |  |  |  |
| 0 | Vvi-Vitvi16g04474\_t001 |  |  |  |  |  |  |  |  |  |  |  |  |
| 0 | Vvi-Vitvi16g04475\_t001 |  |  |  |  |  |  |  |  |  |  |  |  |
| 0 | Vvi-Vitvi16g04476\_t001 |  |  |  |  |  |  |  |  |  |  |  |  |
| 0 | Vvi-Vitvi16g02040\_t001 |  |  |  |  |  |  |  |  |  |  |  |  |
| 0 | Vvi-Vitvi16g04477\_t001 |  |  |  |  |  |  |  |  |  |  |  |  |
| 0 | Vvi-Vitvi16g01279\_t001 |  |  |  |  |  |  |  |  |  |  |  |  |
| 0 | Vvi-Vitvi16g01280\_t001 |  |  |  |  |  |  |  |  |  |  |  |  |
| 1 | Vvi-Vitvi16g01281\_t001 |  | Atr-ERN14141 |  |  |  |  |  |  |  |  |  |  |
| 1 | Vvi-Vitvi16g01282\_t003 |  | Atr-ERN14138 |  |  |  |  |  |  |  |  |  |  |
| 1 | Vvi-Vitvi16g01283\_t001 |  | Atr-ERN14137 |  |  |  |  |  |  |  |  |  |  |
| 1 | Vvi-Vitvi16g01284\_t001 |  | Atr-ERN14135 |  |  |  |  |  |  |  |  |  |  |
| 1 | Vvi-Vitvi16g01285\_t002 |  | Atr-ERN14134 |  |  |  |  |  |  |  |  |  |  |
| 1 | Vvi-Vitvi16g01286\_t001 |  | Atr-ERN14131 |  |  |  |  |  |  |  |  |  |  |
| 1 | Vvi-Vitvi16g04478\_t001 |  | \| \| |  |  |  |  |  |  |  |  |  |  |
| 1 | Vvi-Vitvi16g04479\_t001 |  | \| \| |  |  |  |  |  |  |  |  |  |  |
| 1 | Vvi-Vitvi16g04480\_t001 |  | \| \| |  |  |  |  |  |  |  |  |  |  |
| 1 | Vvi-Vitvi16g04481\_t001 |  | \| \| |  |  |  |  |  |  |  |  |  |  |
| 1 | Vvi-Vitvi16g04482\_t001 |  | \| \| |  |  |  |  |  |  |  |  |  |  |
| 1 | Vvi-Vitvi16g04483\_t001 |  | \| \| |  |  |  |  |  |  |  |  |  |  |
| 1 | Vvi-Vitvi16g04484\_t001 |  | \| \| |  |  |  |  |  |  |  |  |  |  |
| 1 | Vvi-Vitvi16g04485\_t001 |  | \| \| |  |  |  |  |  |  |  |  |  |  |
| 1 | Vvi-Vitvi16g04486\_t001 |  | \| \| |  |  |  |  |  |  |  |  |  |  |
| 1 | Vvi-Vitvi16g01288\_t001 |  | Atr-ERN14130 |  |  |  |  |  |  |  |  |  |  |
| 1 | Vvi-Vitvi16g01289\_t001 |  | Atr-ERN14124 |  |  |  |  |  |  |  |  |  |  |
| 1 | Vvi-Vitvi16g01290\_t002 |  | \| \| |  |  |  |  |  |  |  |  |  |  |
| 1 | Vvi-Vitvi16g01291\_t001 |  | Atr-ERN14120 |  |  |  |  |  |  |  |  |  |  |
| 1 | Vvi-Vitvi16g04487\_t001 |  | \| \| |  |  |  |  |  |  |  |  |  |  |
| 1 | Vvi-Vitvi16g04488\_t001 |  | \| \| |  |  |  |  |  |  |  |  |  |  |
| 1 | Vvi-Vitvi16g01296\_t001 |  | \| \| |  |  |  |  |  |  |  |  |  |  |
| 1 | Vvi-Vitvi16g01297\_t001 |  | Atr-ERN14118 |  |  |  |  |  |  |  |  |  |  |
| 0 | Vvi-Vitvi16g01298\_t001 |  |  |  |  |  |  |  |  |  |  |  |  |
| 0 | Vvi-Vitvi16g04489\_t001 |  |  |  |  |  |  |  |  |  |  |  |  |
| 0 | Vvi-Vitvi16g04490\_t001 |  |  |  |  |  |  |  |  |  |  |  |  |
| 0 | Vvi-Vitvi16g01302\_t001 |  |  |  |  |  |  |  |  |  |  |  |  |
| 0 | Vvi-Vitvi16g04491\_t001 |  |  |  |  |  |  |  |  |  |  |  |  |
| 0 | Vvi-Vitvi16g02049\_t001 |  |  |  |  |  |  |  |  |  |  |  |  |
| 0 | Vvi-Vitvi16g04492\_t001 |  |  |  |  |  |  |  |  |  |  |  |  |
| 0 | Vvi-Vitvi16g02051\_t001 |  |  |  |  |  |  |  |  |  |  |  |  |
| 0 | Vvi-Vitvi16g01303\_t001 |  |  |  |  |  |  |  |  |  |  |  |  |
| 0 | Vvi-Vitvi16g02052\_t001 |  |  |  |  |  |  |  |  |  |  |  |  |
| 0 | Vvi-Vitvi16g04493\_t001 |  |  |  |  |  |  |  |  |  |  |  |  |
| 0 | Vvi-Vitvi16g02053\_t001 |  |  |  |  |  |  |  |  |  |  |  |  |
| 0 | Vvi-Vitvi16g04494\_t001 |  |  |  |  |  |  |  |  |  |  |  |  |
| 0 | Vvi-Vitvi16g04495\_t001 |  |  |  |  |  |  |  |  |  |  |  |  |
| 1 | Vvi-Vitvi16g01307\_t001 |  | Atr-ERN08205 |  |  |  |  |  |  |  |  |  |  |
| 1 | Vvi-Vitvi16g01308\_t001 |  | Atr-ERN08204 |  |  |  |  |  |  |  |  |  |  |
| 1 | Vvi-Vitvi16g01309\_t001 |  | Atr-ERN08199 |  |  |  |  |  |  |  |  |  |  |
| 1 | Vvi-Vitvi16g04496\_t001 |  | \| \| |  |  |  |  |  |  |  |  |  |  |
| 1 | Vvi-Vitvi16g01310\_t001 |  | Atr-ERN08194 |  |  |  |  |  |  |  |  |  |  |
| 1 | Vvi-Vitvi16g01311\_t001 |  | \| \| |  |  |  |  |  |  |  |  |  |  |
| 1 | Vvi-Vitvi16g04497\_t001 |  | \| \| |  |  |  |  |  |  |  |  |  |  |
| 1 | Vvi-Vitvi16g01312\_t001 |  | \| \| |  |  |  |  |  |  |  |  |  |  |
| 1 | Vvi-Vitvi16g01313\_t001 |  | \| \| |  |  |  |  |  |  |  |  |  |  |
| 1 | Vvi-Vitvi16g01314\_t001 |  | \| \| |  |  |  |  |  |  |  |  |  |  |
| 1 | Vvi-Vitvi16g04498\_t001 |  | \| \| |  |  |  |  |  |  |  |  |  |  |
| 1 | Vvi-Vitvi16g02055\_t001.1.6037826c |  | Atr-ERN08189 |  |  |  |  |  |  |  |  |  |  |
| 1 | Vvi-Vitvi16g01316\_t001 |  | Atr-ERN08188 |  |  |  |  |  |  |  |  |  |  |
| 1 | Vvi-Vitvi16g01320\_t001 |  | Atr-ERN08182 |  |  |  |  |  |  |  |  |  |  |
| 1 | Vvi-Vitvi16g04499\_t001 |  | \| \| |  |  |  |  |  |  |  |  |  |  |
| 1 | Vvi-Vitvi16g01322\_t001 |  | \| \| |  |  |  |  |  |  |  |  |  |  |
| 1 | Vvi-Vitvi16g01323\_t001 |  | \| \| |  |  |  |  |  |  |  |  |  |  |
| 1 | Vvi-Vitvi16g01324\_t001 |  | Atr-ERN08156 |  |  |  |  |  |  |  |  |  |  |
| 1 | Vvi-Vitvi16g02056\_t001 |  | \| \| |  |  |  |  |  |  |  |  |  |  |
| 1 | Vvi-Vitvi16g01325\_t001 |  | \| \| |  |  |  |  |  |  |  |  |  |  |
| 1 | Vvi-Vitvi16g01326\_t001 |  | Atr-ERN08144 |  |  |  |  |  |  |  |  |  |  |
| 1 | Vvi-Vitvi16g01327\_t002 |  | Atr-ERN08118 |  |  |  |  |  |  |  |  |  |  |
| 1 | Vvi-Vitvi16g02057\_t001 |  | Atr-ERN08116 |  |  |  |  |  |  |  |  |  |  |
| 1 | Vvi-Vitvi16g02058\_t001 |  | \| \| |  |  |  |  |  |  |  |  |  |  |
| 1 | Vvi-Vitvi16g02059\_t001 |  | Atr-ERN08115 |  |  |  |  |  |  |  |  |  |  |
| 1 | Vvi-Vitvi16g01328\_t001 |  | Atr-ERN08112 |  |  |  |  |  |  |  |  |  |  |
| 1 | Vvi-Vitvi16g01330\_t002 |  | Atr-ERN08105 |  |  |  |  |  |  |  |  |  |  |
| 1 | Vvi-Vitvi16g01331\_t001 |  | Atr-ERN08099 |  |  |  |  |  |  |  |  |  |  |
| 1 | Vvi-Vitvi16g01332\_t001 |  | \| \| |  |  |  |  |  |  |  |  |  |  |
| 1 | Vvi-Vitvi16g01333\_t001 |  | Atr-ERN08098 |  |  |  |  |  |  |  |  |  |  |
| 0 | Vvi-Vitvi16g02060\_t001 |  |  |  |  |  |  |  |  |  |  |  |  |
| 1 | Vvi-Vitvi16g01334\_t001 |  | Atr-ERM96632 |  |  |  |  |  |  |  |  |  |  |
| 1 | Vvi-Vitvi16g02061\_t001 |  | \| \| |  |  |  |  |  |  |  |  |  |  |
| 1 | Vvi-Vitvi16g01336\_t001 |  | \| \| |  |  |  |  |  |  |  |  |  |  |
| 1 | Vvi-Vitvi16g01337\_t001 |  | Atr-ERM96623 |  |  |  |  |  |  |  |  |  |  |
| 1 | Vvi-Vitvi16g02062\_t001 |  | Atr-ERM96622 |  |  |  |  |  |  |  |  |  |  |
| 1 | Vvi-Vitvi16g04500\_t001 |  | \| \| |  |  |  |  |  |  |  |  |  |  |
| 1 | Vvi-Vitvi16g04501\_t001 |  | \| \| |  |  |  |  |  |  |  |  |  |  |
| 1 | Vvi-Vitvi16g04502\_t001 |  | \| \| |  |  |  |  |  |  |  |  |  |  |
| 1 | Vvi-Vitvi16g01338\_t001 |  | Atr-ERM96618 |  |  |  |  |  |  |  |  |  |  |
| 1 | Vvi-Vitvi16g01339\_t001 |  | Atr-ERM96616 |  |  |  |  |  |  |  |  |  |  |
| 1 | Vvi-Vitvi16g01340\_t001 |  | Atr-ERM96614 |  |  |  |  |  |  |  |  |  |  |
| 0 | Vvi-Vitvi16g04503\_t001 |  |  |  |  |  |  |  |  |  |  |  |  |
| 0 | Vvi-Vitvi16g01341\_t001 |  |  |  |  |  |  |  |  |  |  |  |  |
| 0 | Vvi-Vitvi16g01342\_t001 |  |  |  |  |  |  |  |  |  |  |  |  |
| 0 | Vvi-Vitvi16g04504\_t001 |  |  |  |  |  |  |  |  |  |  |  |  |
| 0 | Vvi-Vitvi16g01344\_t002 |  |  |  |  |  |  |  |  |  |  |  |  |
| 0 | Vvi-Vitvi16g04505\_t001 |  |  |  |  |  |  |  |  |  |  |  |  |
| 0 | Vvi-Vitvi16g01346\_t001 |  |  |  |  |  |  |  |  |  |  |  |  |
| 0 | Vvi-Vitvi16g01347\_t001 |  |  |  |  |  |  |  |  |  |  |  |  |
| 0 | Vvi-Vitvi16g02066\_t001 |  |  |  |  |  |  |  |  |  |  |  |  |
| 0 | Vvi-Vitvi16g02067\_t001 |  |  |  |  |  |  |  |  |  |  |  |  |
| 0 | Vvi-Vitvi16g04506\_t001 |  |  |  |  |  |  |  |  |  |  |  |  |
| 0 | Vvi-Vitvi16g02068\_t001 |  |  |  |  |  |  |  |  |  |  |  |  |
| 0 | Vvi-Vitvi16g01348\_t005 |  |  |  |  |  |  |  |  |  |  |  |  |
| 0 | Vvi-Vitvi16g01349\_t001 |  |  |  |  |  |  |  |  |  |  |  |  |
| 0 | Vvi-Vitvi16g04507\_t001 |  |  |  |  |  |  |  |  |  |  |  |  |
| 0 | Vvi-Vitvi16g01350\_t001 |  |  |  |  |  |  |  |  |  |  |  |  |
| 0 | Vvi-Vitvi16g02069\_t001 |  |  |  |  |  |  |  |  |  |  |  |  |
| 0 | Vvi-Vitvi16g02070\_t001 |  |  |  |  |  |  |  |  |  |  |  |  |
| 0 | Vvi-Vitvi16g01352\_t001 |  |  |  |  |  |  |  |  |  |  |  |  |
| 0 | Vvi-Vitvi16g01353\_t001 |  |  |  |  |  |  |  |  |  |  |  |  |
| 0 | Vvi-Vitvi16g04508\_t001 |  |  |  |  |  |  |  |  |  |  |  |  |
| 0 | Vvi-Vitvi16g01354\_t001 |  |  |  |  |  |  |  |  |  |  |  |  |
| 0 | Vvi-Vitvi16g04509\_t001 |  |  |  |  |  |  |  |  |  |  |  |  |
| 0 | Vvi-Vitvi16g01355\_t001 |  |  |  |  |  |  |  |  |  |  |  |  |
| 0 | Vvi-Vitvi16g01356\_t001 |  |  |  |  |  |  |  |  |  |  |  |  |
| 0 | Vvi-Vitvi16g01357\_t001 |  |  |  |  |  |  |  |  |  |  |  |  |
| 0 | Vvi-Vitvi16g04510\_t001 |  |  |  |  |  |  |  |  |  |  |  |  |
| 0 | Vvi-Vitvi16g02071\_t001 |  |  |  |  |  |  |  |  |  |  |  |  |
| 0 | Vvi-Vitvi16g01358\_t001 |  |  |  |  |  |  |  |  |  |  |  |  |
| 1 | Vvi-Vitvi16g01359\_t001 |  | Atr-ERN10762 |  |  |  |  |  |  |  |  |  |  |
| 2 | Vvi-Vitvi16g01360\_t001 |  | Atr-ERN10755 |  | Atr-ERN10755 |  |  |  |  |  |  |  |  |
| 2 | Vvi-Vitvi16g02072\_t002 |  | \| \| |  | \| \| |  |  |  |  |  |  |  |  |
| 2 | Vvi-Vitvi16g04511\_t001 |  | \| \| |  | \| \| |  |  |  |  |  |  |  |  |
| 2 | Vvi-Vitvi16g01362\_t001 |  | Atr-ERN10742 |  | \| \| |  |  |  |  |  |  |  |  |
| 2 | Vvi-Vitvi16g01363\_t001 |  | Atr-ERN10739 |  | \| \| |  |  |  |  |  |  |  |  |
| 2 | Vvi-Vitvi16g01364\_t001 |  | Atr-ERN10736 |  | \| \| |  |  |  |  |  |  |  |  |
| 2 | Vvi-Vitvi16g01365\_t001 |  | Atr-ERN10723 |  | \| \| |  |  |  |  |  |  |  |  |
| 2 | Vvi-Vitvi16g01366\_t002 |  | Atr-ERN10721 |  | \| \| |  |  |  |  |  |  |  |  |
| 2 | Vvi-Vitvi16g01367\_t001 |  | Atr-ERN10712 |  | \| \| |  |  |  |  |  |  |  |  |
| 2 | Vvi-Vitvi16g01370\_t001 |  | \| \| |  | \| \| |  |  |  |  |  |  |  |  |
| 2 | Vvi-Vitvi16g04512\_t001 |  | \| \| |  | \| \| |  |  |  |  |  |  |  |  |
| 2 | Vvi-Vitvi16g01371\_t001 |  | Atr-ERN10702 |  | \| \| |  |  |  |  |  |  |  |  |
| 1 | Vvi-Vitvi16g04513\_t001 |  |  |  | \| \| |  |  |  |  |  |  |  |  |
| 1 | Vvi-Vitvi16g01373\_t001 |  |  |  | Atr-ERN10765 |  |  |  |  |  |  |  |  |
| 1 | Vvi-Vitvi16g01374\_t001 |  |  |  | Atr-ERN10771 |  |  |  |  |  |  |  |  |
| 1 | Vvi-Vitvi16g01375\_t001 |  |  |  | \| \| |  |  |  |  |  |  |  |  |
| 1 | Vvi-Vitvi16g02073\_t001 |  |  |  | Atr-ERN10777 |  |  |  |  |  |  |  |  |
| 1 | Vvi-Vitvi16g02074\_t001 |  |  |  | Atr-ERN10780 |  |  |  |  |  |  |  |  |
| 1 | Vvi-Vitvi16g02075\_t001 |  |  |  | Atr-ERN10785 |  |  |  |  |  |  |  |  |
| 0 | Vvi-Vitvi16g01377\_t001 |  |  |  |  |  |  |  |  |  |  |  |  |
| 0 | Vvi-Vitvi16g01379\_t001 |  |  |  |  |  |  |  |  |  |  |  |  |
| 0 | Vvi-Vitvi16g04514\_t001 |  |  |  |  |  |  |  |  |  |  |  |  |
| 0 | Vvi-Vitvi16g01380\_t001 |  |  |  |  |  |  |  |  |  |  |  |  |
| 0 | Vvi-Vitvi16g02077\_t003 |  |  |  |  |  |  |  |  |  |  |  |  |
| 0 | Vvi-Vitvi16g04515\_t001 |  |  |  |  |  |  |  |  |  |  |  |  |
| 0 | Vvi-Vitvi16g01382\_t001 |  |  |  |  |  |  |  |  |  |  |  |  |
| 0 | Vvi-Vitvi16g01383\_t001 |  |  |  |  |  |  |  |  |  |  |  |  |
| 0 | Vvi-Vitvi16g02078\_t001 |  |  |  |  |  |  |  |  |  |  |  |  |
| 0 | Vvi-Vitvi16g01384\_t001 |  |  |  |  |  |  |  |  |  |  |  |  |
| 0 | Vvi-Vitvi16g01386\_t001 |  |  |  |  |  |  |  |  |  |  |  |  |
| 0 | Vvi-Vitvi16g02079\_t001 |  |  |  |  |  |  |  |  |  |  |  |  |
| 0 | Vvi-Vitvi16g01387\_t001 |  |  |  |  |  |  |  |  |  |  |  |  |
| 0 | Vvi-Vitvi16g04516\_t001 |  |  |  |  |  |  |  |  |  |  |  |  |
| 0 | Vvi-Vitvi16g02080\_t001 |  |  |  |  |  |  |  |  |  |  |  |  |
| 0 | Vvi-Vitvi16g01388\_t001 |  |  |  |  |  |  |  |  |  |  |  |  |
| 0 | Vvi-Vitvi16g01389\_t001 |  |  |  |  |  |  |  |  |  |  |  |  |
| 0 | Vvi-Vitvi16g01390\_t001 |  |  |  |  |  |  |  |  |  |  |  |  |
| 0 | Vvi-Vitvi16g01391\_t001 |  |  |  |  |  |  |  |  |  |  |  |  |
| 0 | Vvi-Vitvi16g02081\_t001 |  |  |  |  |  |  |  |  |  |  |  |  |
| 0 | Vvi-Vitvi16g04517\_t003 |  |  |  |  |  |  |  |  |  |  |  |  |
| 0 | Vvi-Vitvi16g01393\_t002 |  |  |  |  |  |  |  |  |  |  |  |  |
| 0 | Vvi-Vitvi16g01394\_t001 |  |  |  |  |  |  |  |  |  |  |  |  |
| 0 | Vvi-Vitvi16g02084\_t001 |  |  |  |  |  |  |  |  |  |  |  |  |
| 0 | Vvi-Vitvi16g01395\_t001 |  |  |  |  |  |  |  |  |  |  |  |  |
| 0 | Vvi-Vitvi16g01396\_t001 |  |  |  |  |  |  |  |  |  |  |  |  |
| 0 | Vvi-Vitvi16g04518\_t001 |  |  |  |  |  |  |  |  |  |  |  |  |
| 0 | Vvi-Vitvi16g01398\_t001 |  |  |  |  |  |  |  |  |  |  |  |  |
| 1 | Vvi-Vitvi16g02085\_t001 |  | Atr-ERN14055 |  |  |  |  |  |  |  |  |  |  |
| 1 | Vvi-Vitvi16g01399\_t002 |  | Atr-ERN14056 |  |  |  |  |  |  |  |  |  |  |
| 1 | Vvi-Vitvi16g04519\_t002 |  | Atr-ERN14057 |  |  |  |  |  |  |  |  |  |  |
| 1 | Vvi-Vitvi16g01401\_t001.2.6037826c |  | Atr-ERN14058 |  |  |  |  |  |  |  |  |  |  |
| 1 | Vvi-Vitvi16g01402\_t001 |  | Atr-ERN14061 |  |  |  |  |  |  |  |  |  |  |
| 1 | Vvi-Vitvi16g02087\_t001 |  | \| \| |  |  |  |  |  |  |  |  |  |  |
| 1 | Vvi-Vitvi16g01403\_t002 |  | Atr-ERN14063 |  |  |  |  |  |  |  |  |  |  |
| 1 | Vvi-Vitvi16g01404\_t001 |  | Atr-ERN14072 |  |  |  |  |  |  |  |  |  |  |
| 1 | Vvi-Vitvi16g02088\_t001 |  | \| \| |  |  |  |  |  |  |  |  |  |  |
| 1 | Vvi-Vitvi16g01405\_t001 |  | Atr-ERN14077 |  |  |  |  |  |  |  |  |  |  |
| 1 | Vvi-Vitvi16g01406\_t001 |  | Atr-ERN14080 |  |  |  |  |  |  |  |  |  |  |
| 1 | Vvi-Vitvi16g04520\_t001 |  | \| \| |  |  |  |  |  |  |  |  |  |  |
| 1 | Vvi-Vitvi16g02089\_t001 |  | \| \| |  |  |  |  |  |  |  |  |  |  |
| 1 | Vvi-Vitvi16g04521\_t001 |  | \| \| |  |  |  |  |  |  |  |  |  |  |
| 1 | Vvi-Vitvi16g01407\_t001 |  | Atr-ERN14083 |  |  |  |  |  |  |  |  |  |  |
| 1 | Vvi-Vitvi16g01409\_t001 |  | \| \| |  |  |  |  |  |  |  |  |  |  |
| 1 | Vvi-Vitvi16g01410\_t001 |  | Atr-ERN14091 |  |  |  |  |  |  |  |  |  |  |
| 1 | Vvi-Vitvi16g01411\_t001 |  | Atr-ERN14103 |  |  |  |  |  |  |  |  |  |  |
| 1 | Vvi-Vitvi16g01412\_t001 |  | \| \| |  |  |  |  |  |  |  |  |  |  |
| 1 | Vvi-Vitvi16g01413\_t001 |  | \| \| |  |  |  |  |  |  |  |  |  |  |
| 1 | Vvi-Vitvi16g01415\_t001 |  | Atr-ERN14106 |  |  |  |  |  |  |  |  |  |  |
| 1 | Vvi-Vitvi16g04522\_t001 |  | \| \| |  |  |  |  |  |  |  |  |  |  |
| 1 | Vvi-Vitvi16g01416\_t002 |  | Atr-ERN14110 |  |  |  |  |  |  |  |  |  |  |
| 1 | Vvi-Vitvi16g01417\_t003 |  | Atr-ERN14111 |  |  |  |  |  |  |  |  |  |  |
| 1 | Vvi-Vitvi16g01418\_t001 |  | Atr-ERN14116 |  |  |  |  |  |  |  |  |  |  |
| 0 | Vvi-Vitvi16g04523\_t001 |  |  |  |  |  |  |  |  |  |  |  |  |
| 0 | Vvi-Vitvi16g01419\_t001 |  |  |  |  |  |  |  |  |  |  |  |  |
